# SLIT2 as a key regulator and therapeutic target in liver injury

**DOI:** 10.1016/j.ymthe.2026.01.010

**Published:** 2026-01-10

**Authors:** Yong Won Choi, Jae Ho Choi, Young-Sam Lee, Jinju Jeong, Eunho Kang, So Hyun Park, Young-Kyoung Lee, Soon Sang Park, Hee Young Kang, Young Hwa Kim, Tae Jun Park

**Affiliations:** 1 Inflamm-Aging Translational Research Center, Ajou University Medical Center, Suwon 16499, Korea; 2 Department of New Biology, Daegu Gyeongbuk Institute of Science & Technology, Daegu 42988, Korea; 3 Department of Pathology, Ajou University School of Medicine, Suwon 16499, Korea; 4 Department of Hematology-Oncology, Ajou University School of Medicine, Suwon 16499, Korea; 5 Department of Biochemistry and Molecular Biology, Ajou University School of Medicine, Suwon 16499, Korea; 6 Department of Biomedical Sciences, Ajou University Graduate School of Medicine, Suwon 16499, Korea; 7 Department of Dermatology, Ajou University School of Medicine, Suwon 16499, Korea; 8 Molecular Neurobiology Laboratory, McLean Hospital/Harvard Medical School, Belmont, MA 02478, USA

**Keywords:** slit2, liver injury, oxidative stress, CYP2E1, Robo4

## Abstract

Drug-induced liver injury accounts for approximately 10% of acute hepatitis and up to 50% of acute liver failure. Despite its clinical significance, treatment remains largely limited to cessation of the offending agent. SLIT/ROBO signaling, known for roles in organ development, angiogenesis, leukocyte migration, and cancer metastasis, has demonstrated protective effects against various organ damage. In mouse models of liver injury induced by acetaminophen (APAP), thioacetamide, bile duct ligation, and serum from patients with toxic liver disease, *Slit2* expression significantly increases, while *Slit1* and *Slit3* remain unchanged. Liver-specific *Slit2* knockdown exacerbates liver injury, whereas recombinant SLIT2 alleviates liver damage by reducing oxidative stress via CYP2E1 downregulation and suppressing inflammation through nuclear factor κB inhibition. Notably, among ROBO receptors, only ROBO4 was induced in hepatocytes after APAP exposure. ROBO4 knockdown eliminates the hepatoprotective effects of SLIT2, highlighting the importance of SLIT2/BOBO4 signaling in toxic liver injury. Furthermore, the novel Slit2-derived peptide 5 (SP5), designed from the ROBO4-binding LRR2 domain, significantly reduces liver damage and inflammation. Notably, both recombinant SLIT2 and SP5 confer hepatoprotection even when administered 24 h after APAP challenge. These findings suggest that SLIT2/ROBO4-targeted therapies may offer a promising approach for preventing fulminant hepatitis in the context of toxic liver injury.

## Graphical abstract

**Figure:**
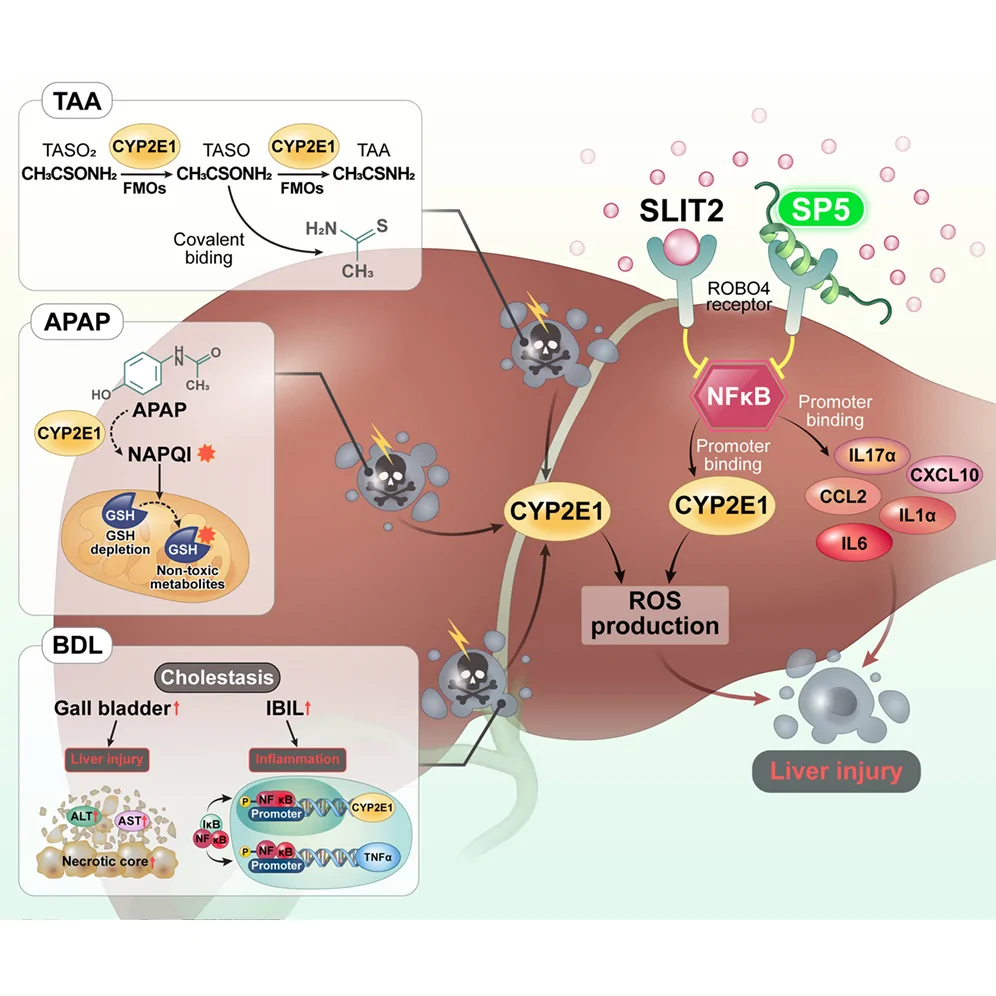

## Teaser

Kim and colleagues identify SLIT2/ROBO4 signaling as a protective pathway in toxic liver injury. They show that recombinant SLIT2 and a novel SLIT2-derived peptide reduce CYP2E1-driven oxidative stress and inflammation, highlighting this axis as a promising therapeutic target for toxic hepatitis.

## Introduction

The liver is a major organ responsible for metabolic and detoxification processes, making it particularly susceptible to the production and exposure to reactive oxygen species (ROS).^1^ Oxidative stress, resulting from the disruption of redox balance, is a fundamental pathological mechanism in various liver diseases, including drug-induced liver injury, chronic viral hepatitis B and C, alcoholic fatty liver disease, non-alcoholic fatty liver disease, and steatohepatitis.^2^ Intracellular ROS can be generated through the mitochondrial respiratory chain or peroxisomes, or from enzymatic reactions involving xanthine oxidases, NADPH oxidases, and cytochrome P450 (CYP) oxidases. Among them, CYPs are predominantly expressed in liver hepatocytes and are involved in drug and xenobiotic metabolism as well as biosynthesis of endogenous molecules. However, CYP enzymes can also contribute to ROS production through uncoupling of their enzymatic cycle.^3^ Among the 57 CYP genes in the human genome, CYP2E1 is well known as a unique “leaky” enzyme due to the constitutively high-spin state of the heme iron, which allows direct reduction of molecular oxygen, thereby generating high capacity for oxyradical production.^4^ Moreover, CYP2E1 metabolizes various carcinogenic compounds, including chloroform and benzene as well as drugs such as acetaminophen (APAP), salicylic acid, and several inhalational anesthetics, converting them into toxic intermediates. Accumulating evidence suggests that CYP2E1 plays a significant role in the pathogenesis of both acute and chronic liver diseases induced by drugs, toxic agents, and alcohol abuse, as well as in the progression of simple fatty liver to non-alcoholic steatohepatitis.^5^ Interestingly, previous studies have demonstrated that aged CYP2E1-null mice exhibit reduced aging-related pathological changes in the liver^6^ and kidney,^7^ suggesting a potential role for endogenous CYP2E1 in liver and kidney aging. Despite the critical role of CYP2E1 in various hepatic pathophysiological conditions, efforts to develop clinically available CYP2E1 inhibitors have been largely unsuccessful. Currently, *N*-acetylcysteine (NAC) is the only clinically effective treatment for hepatotoxicity induced by APAP and other causes,^8^ although its efficacy is limited to early administration within 6–12 h. Therefore, identifying novel molecular targets to ameliorate liver injury of various etiologies remains a significant unmet medical need.

The SLIT/Roundabout (ROBO) signaling axis is an evolutionarily conserved pathway composed of secretory SLIT proteins (three isoforms, SLIT1–3) and their corresponding receptor ROBO (four isoforms, ROBO1–4).^9^ Initially identified for its role in axonal guidance across the midline during brain development,^10^ SLIT/ROBO signaling has since been implicated in a wide array of biological processes including organ development, angiogenesis, leukocyte migration, and cancer metastasis.^11^ Interestingly, beyond its developmental roles in the brain, kidney,^12^ heart,^13^ and vasculature,^14^ SLIT2 has been consistently shown to exert protective effects against various forms of organ damage. For instance, SLIT2 has demonstrated anti-inflammatory and anti-oxidative properties in multiple models of injury, including neuroinflammation induced by germinal matrix hemorrhage,^15^ surgical brain injury,^16^ acute hypoxic kidney injury,^17^ coronary heart disease,^18^ and lipopolysaccharide-induced endothelial inflammation.^19^ Additionally, we also identified anti-inflammatory properties and rejuvenating potential of SLIT2 in mid-old fibroblasts and aged mice.^20^ Increased SLIT2 levels have been observed in liver-damaged conditions such as liver fibrosis in human patients and CCl4-induced liver fibrosis in a mouse model. However, in contrast to anti-fibrotic activity of SLIT2 during kidney injury,^21^ SLIT2 transgenic mice were more susceptible to CCl4-induced liver injury and fibrosis by activating hepatic stellate cells.^22^ Therefore, it would be of great interest to determine whether exogenous SLIT2 can protect against or exacerbate liver damage caused by various acute and subacute insults.

To elucidate the role of the SLIT2/ROBO pathway in liver injury, we employed three distinct models of liver damage: APAP, thioacetamide (TAA), and bile duct ligation (BDL). All three models exhibited increased SLIT2 expression following liver injury. Notably, the administration of exogenous SLIT2, either before or even 24 h after injury, significantly attenuated liver damage. Mechanistically, SLIT2 suppressed the upregulation of CYP2E1 during liver injury by inhibiting nuclear factor κB (NF-κB) transcriptional activity, thereby reducing ROS generation and associated inflammation via the ROBO4 receptor. Peptides capable of activating ROBO4 were designed based on the leucine-rich repeat (LRR2) domain of SLIT2, a region critical for receptor binding. A ROBO4-specific recombinant SLIT2 peptide demonstrated hepatoprotective effects comparable to those of full-length SLIT2 recombinant treatment. These findings suggest that the SLIT2/ROBO4 pathway plays a protective role in various liver injury models and that a ROBO4-specific SLIT2 peptide presents a promising therapeutic strategy for preventing liver damage and the progression of fulminant hepatitis across diverse clinical contexts.

## Results

### Upregulation of SLIT2 expression mediates protective effects against hepatotoxicity in liver injury

**Figure 1:**
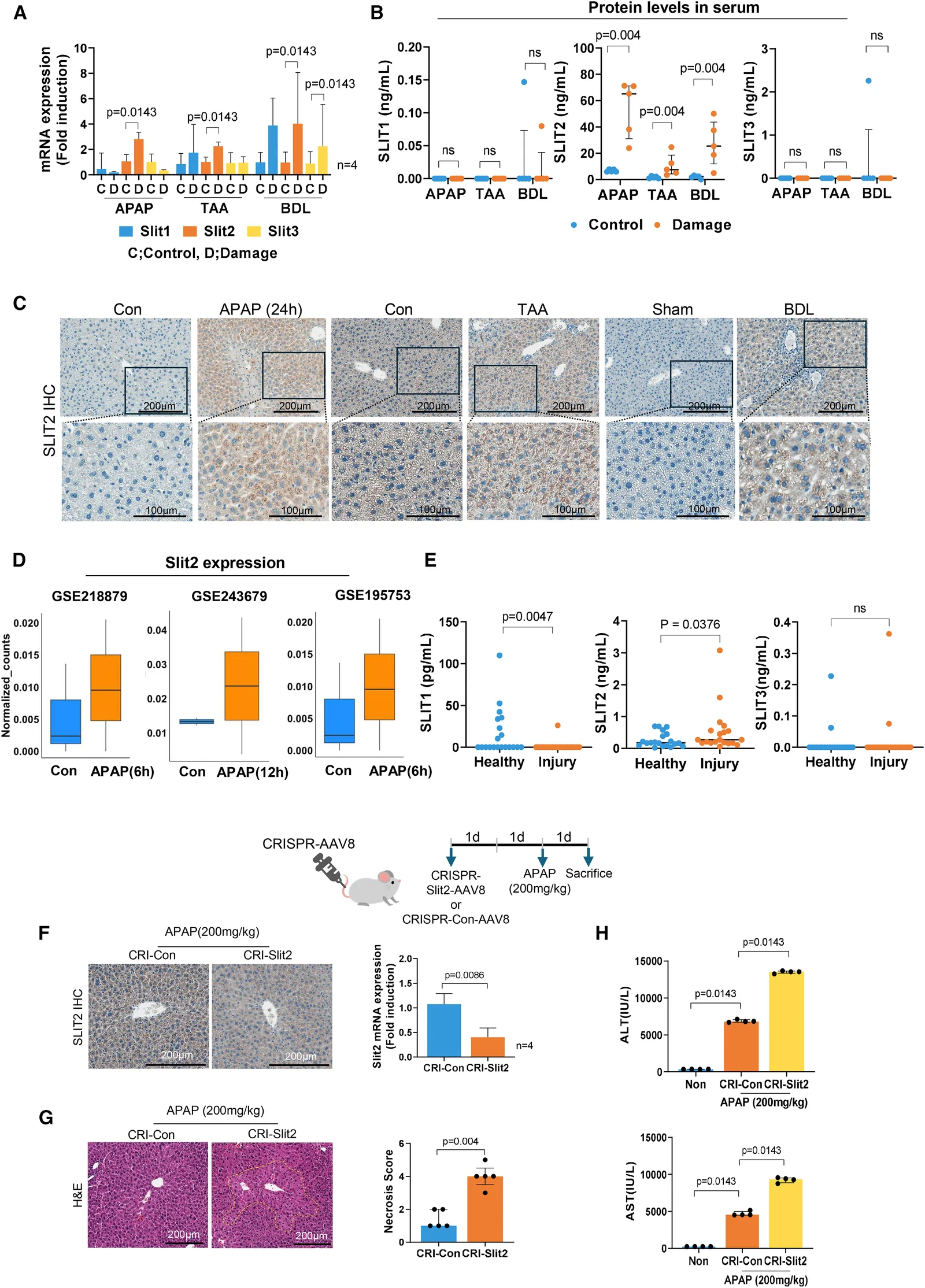
SLIT2 response to liver injury Liver injury was induced in C57BL/6J mice using acetaminophen. (A) *Slit1–3* mRNA expression in liver tissue from APAP-treated, TAA-treated, or BDL mice. (B) ELISA analysis of SLIT1–3 levels in serum from APAP-treated, TAA-treated, or BDL mice (*n* = 5 per group). (C) IHC analysis of SLIT2 expression in liver tissues from each injury model. The “sham” group refers to the non-surgical control group that did not undergo BDL. (D) *Slit2* expression analysis using publicly available mouse liver mRNA datasets. Expression levels were compared between control and APAP-treated groups at 6 h (GEO: GSE218879 and GSE195753) and 12 h (GEO: GSE24367) post treatment. (E) ELISA analysis of serum SLIT1–3 levels in human subjects with toxic liver injury (*n* = 20) and healthy controls (*n* = 20). Statistical significance was determined using Student’s *t* test. (F–H) The upper panel illustrates the experimental scheme. Mice were intravenously injected with CRISPR-Slit2 AAV8 virus (CRI-Slit2) or control virus (CRI-Con), followed by APAP (200 mg/kg) administration. (F) IHC analysis of SLIT2 expression in CRISPR-Slit2 AAV8-injected liver tissue. The right panel shows *Slit2* mRNA expression in liver tissue. (G) H&E staining of liver tissues. The left panel shows representative images; yellow areas indicate necrosis. The right panel shows quantification of necrosis scores. Images were acquired and analyzed from five randomly selected fields per sample. (H) Serum ALT and AST levels in CRI-Con and CRI-Slit2 groups (*n* = 4 per group). The “Non” group represents age-matched mice treated with 1× PBS instead of APAP. Real-time PCR experiments were performed in four independent replicates. The expression profiles in (A)–(C) were derived from the same untreated and injury groups shown in Figure 2, where histological and serological analyses confirmed successful establishment of the liver injury models. Statistical significance, except for (D) and (E), was determined using the Mann-Whitney U test; data are presented as median ± IQR.

To evaluate the potential activation of the Slit/Robo axis in liver injury, we first analyzed the expression levels of its ligands SLIT1, SLIT2, and SLIT3 in mouse liver tissue and serum from acute and subacute liver injury models induced by APAP treatment, TAA treatment, and BDL. Model establishment was verified by hematoxylin and eosin (H&E) staining (Figure S1A) with quantification of necrotic areas (Figure S1B), together with serum alanine aminotransferase (ALT) and aspartate aminotransferase (AST) measurements (Figure S1C). Notably, all three models exhibited a significant increase in *Slit2* mRNA (Figure 1A), serum SLIT2 levels (Figure 1B), and SLIT2 protein levels in liver tissue (Figure 1C). In contrast, *Slit1* and *Slit3* expression remained unchanged (Figures 1A and 1B). To further validate these findings, we analyzed publicly available RNA sequencing (RNA-seq) datasets (GEO: GSE218879, GSE243679, and GSE195753) from APAP-induced liver injury mouse models. Consistently, these datasets demonstrated a significant upregulation of *Slit2* (Figure 1D). However, since no human RNA-seq dataset from toxic liver injury cohorts was available in the literature, we utilized serum samples obtained from a tissue bank at Ajou University Hospital, collected from patients with acute liver injury caused by the ingestion of various toxic substances. Consistent with our findings in mouse models, SLIT2 levels were significantly elevated in the serum of patients with toxic liver injury compared to age-matched healthy controls (Figure 1E). Taken together, these results suggest that SLIT2 is the predominant ligand responsible for activating the SLIT/ROBO axis in the context of toxic liver injury.

To investigate the functional role of increased SLIT2 following liver damage, liver-specific knockdown of *Slit2* was induced using CRISPR-*Slit2*-AAV8 (Figure 1F). Successful knockdown was confirmed by immunohistochemistry (IHC) and real-time PCR (Figure 1F). Histological analysis (Figure 1G) and serum measurements of AST and ALT (Figure 1H) revealed that liver injury was exacerbated in mice with liver-specific *Slit2* knockdown, accompanied by increased necrosis. Upon APAP treatment, the immortalized human hepatic cell line HepaRG and the *TP53* wild-type hepatic carcinoma cell line HepG2 exhibited a dose-dependent increase in *Slit2* mRNA expression (Figure S2A). In HepaRG cell lines, knockdown of *Slit2* using small interfering RNA (siRNA) further aggravated APAP-induced cytotoxicity, as evidenced by TUNEL assay (Figure S2B) and MTT assay (Figure S2C). Collectively, these findings suggest that toxic liver injury in both mice and humans activates the SLIT2/ROBO axis via SLIT2 upregulation and that elevated SLIT2 may play a critical role in mitigating tissue damage.

### SLIT2 treatment mitigates liver injury induced by APAP, TAA, and BDL

**Figure 2:**
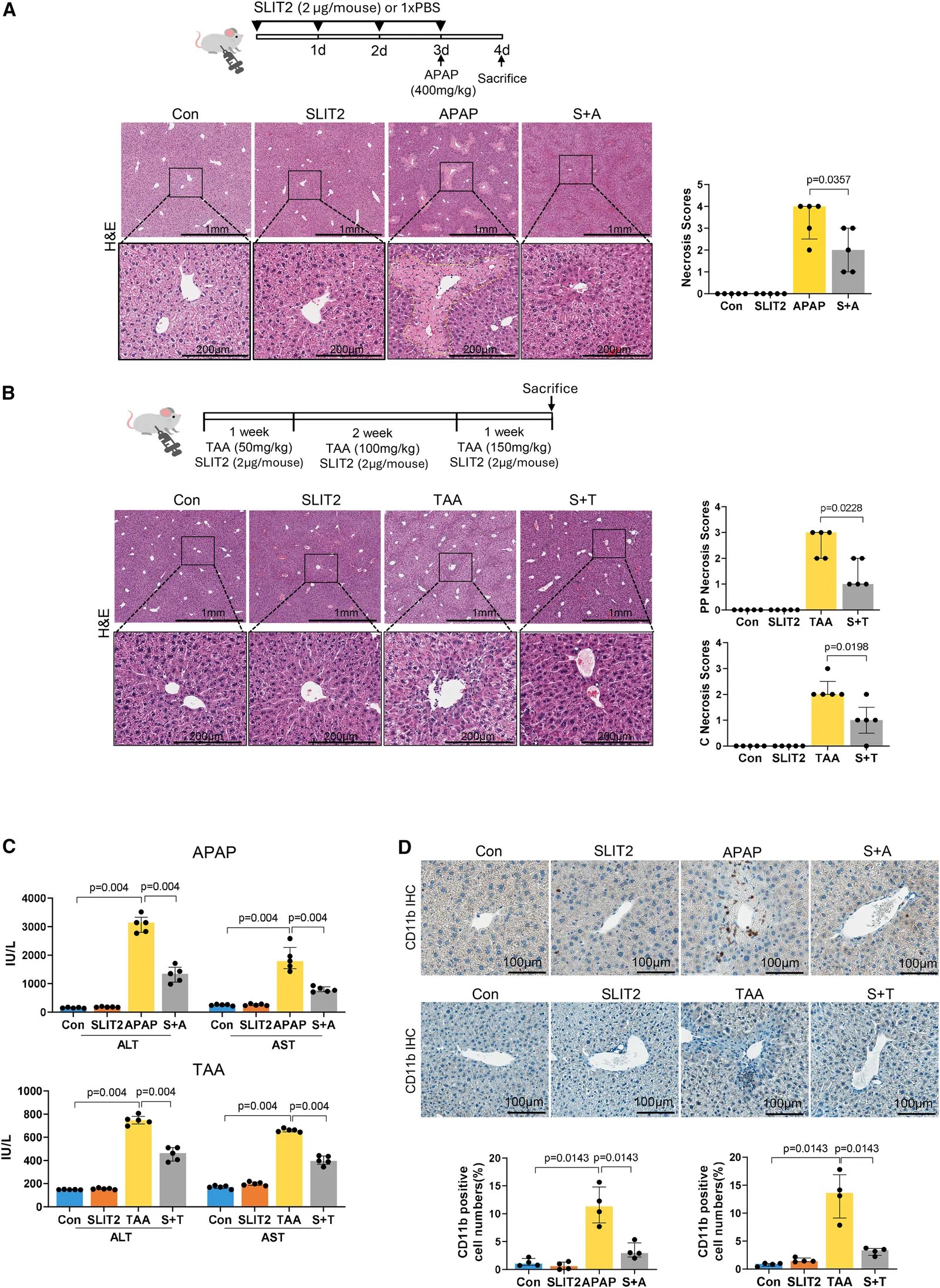
SLIT2 suppresses liver toxicity in mouse liver injury models (A) APAP-induced acute liver injury model. Mice were treated with recombinant mouse SLIT2 (rmSLIT2; 2 μg/mouse) or PBS prior to APAP (400 mg/kg) administration. Representative H&E-stained liver sections are shown; necrotic regions are indicated. Quantification of necrotic areas was performed from five fields per sample (*n* = 5 per group). (B) TAA-induced chronic liver injury model. Mice received TAA (increasing weekly doses) with or without rmSLIT2 (2 μg/mouse, twice weekly). Representative H&E images are shown, with quantification of periportal/periseptal (PP) and confluent (C) necrotic areas (*n* = 5 per group). (C) Serum ALT and AST levels from the APAP model described in (A) (upper panel) and from the TAA model described in (B) (lower panel), respectively (*n* = 5 per group). (D) CD11b-positive immune cell infiltration in APAP- and TAA-induced liver injury. Representative images are shown, and CD11b-positive cells were quantified from four randomly selected fields per condition. Data are presented as median ± IQR. Statistical significance was determined using the Mann-Whitney U test.

To enhance the protective role of SLIT2 in liver injury, recombinant mouse SLIT2 (rmSLIT2) was administered immediately before and after liver damage induced by APAP, TAA, and BDL. A sublethal dose of APAP and TAA induced characteristic histological changes in the liver, including necrosis and fibrosis, while rmSLIT2 treatment significantly attenuated these alterations. H&E staining revealed a reduction in necrosis areas induced by APAP and TAA (Figures 2A and 2B). Consistent with the histological changes, serum AST and ALT elevations were significantly lowered following treatment (Figure 2C). To assess the degree of inflammation, we analyzed CD11b infiltration, which was significantly reduced in the rmSLIT2-treated group (Figure 2D). In the subacute liver injury model induced by BDL, rmSLIT2 treatment mitigated histological damage and reduced serum AST and ALT elevations compared to controls (Figure S3A). CD11b infiltration was also downregulated in the rmSLIT2-treated group (Figure S3B). Furthermore, survival analysis of mice with BDL showed a clear trend toward improved long-term survival in the rmSLIT2-treatment group (Figure S3C). Taken together, these findings demonstrated the hepatoprotective role of SLIT2, supporting its potential as a therapeutic strategy for acute and subacute liver injury.

### SLIT2 reduces ROS generation in toxic liver injury by downregulating CYP2E1 expression

**Figure 3:**
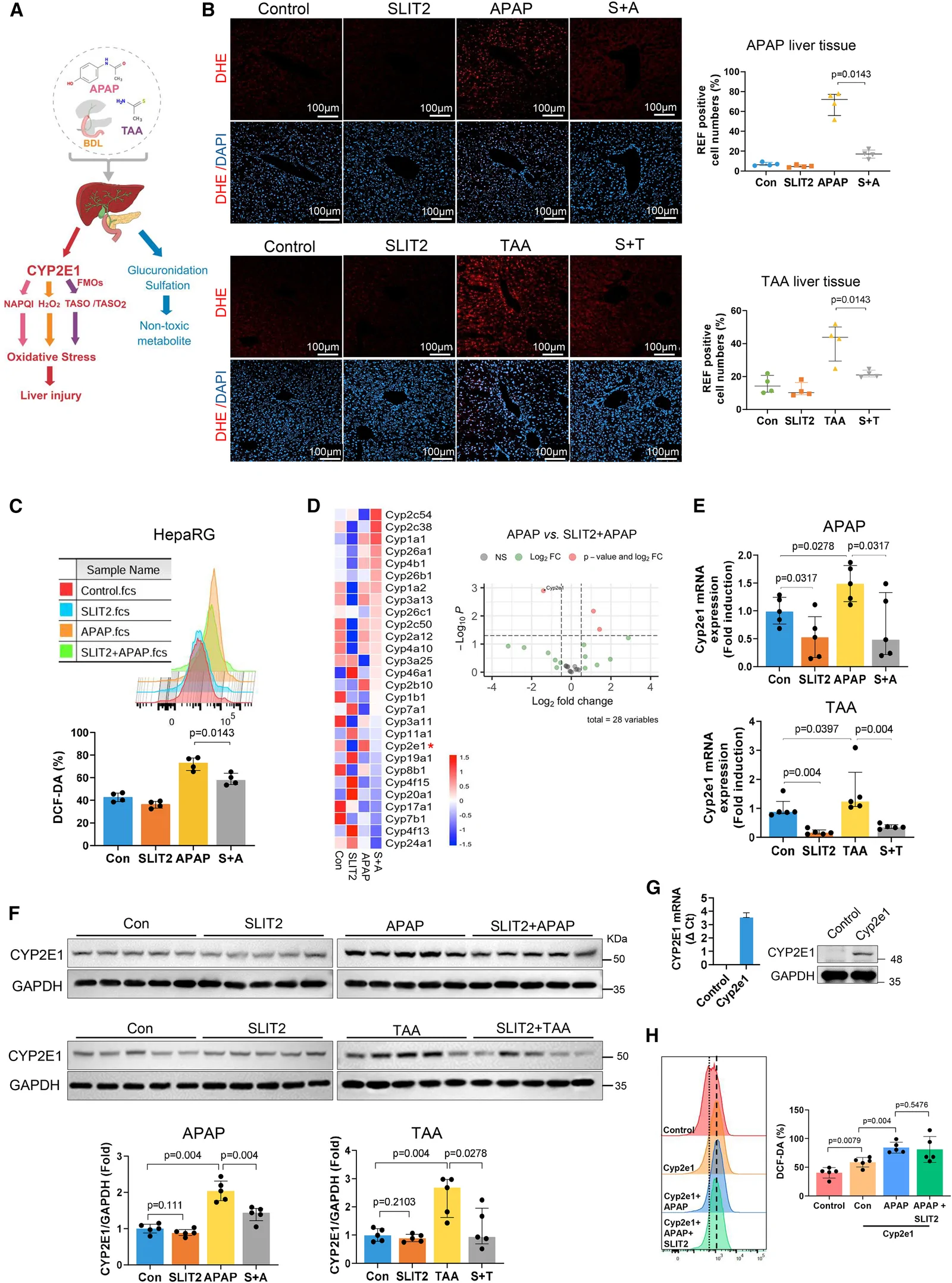
SLIT2 suppresses oxidative stress by regulating CYP2E1 expression (A) Schematic illustration of the liver injury models used in this study. (B) Oxidative stress analysis in liver tissue. Frozen liver tissues from APAP- or TAA-treated mice were stained with dihydroethidium (DHE) and DAPI to assess oxidative stress. DHE-positive cells were quantified from four randomly selected fields per group. (C) ROS generation in HepaRG cells was analyzed by fluorescence-activated cell sorting. Fluorescence shifts in histograms reflect changes in ROS levels under different conditions. The lower panel presents quantification of ROS levels, expressed as mean fluorescence intensity (MFI). (D) Left: heatmap of cytochrome P450 family-related gene expression. Gene-expression profiles were compared among control, rmSLIT2, APAP, and rmSLIT2 + APAP treatment groups. Right: volcano data comparing APAP vs. rmSLIT2 + APAP among the CYP group, the *x* axis represents log_2_ fold change, and the *y* axis represents −log_10_*p* value. Genes highlighted in red meet both statistical significance (*p* < 0.05) and absolute fold-change thresholds (|log_2_FC| > 1). (E) Real-time PCR analysis of *Cyp2e1* mRNA expression in liver tissues from APAP- or TAA-treated mice (*n* = 5 per group). (F) Western blot analysis of CYP2E1 protein expression in liver tissues. Samples from control (Con) and rmSLIT2-, APAP-, and rmSLIT2 + APAP-treated groups were analyzed (*n* = 5 per group). Protein bands were quantified by densitometry, and expression levels were normalized to GAPDH as a loading control. (G and H) CYP2E1 overexpression in empty vector control and Cyp2e1-transfected cells. Cells were transfected with empty vector (Control) or the CYP2E1 overexpression vector (Cyp2e1). After 24 h, cells were treated with 3 mM acetaminophen (APAP) or 100 ng/mL recombinant mouse SLIT2 protein. (G) Real-time PCR and western blot analysis confirming CYP2E1 overexpression. The *y* axis represents CYP2E1 mRNA expression (ΔCt). CYP2E1 transcripts were not detected in the control group, and therefore ΔCt values could be obtained only in the CYP2E1-overexpressing cells. (H) ROS measurement by flow cytometry under the same conditions. Quantification is shown on the right (*n* = 5). Data are presented as median ± IQR. Statistical significance was determined using the Mann-Whitney U test.

APAP and TAA are metabolized by CYP2E1, generating reactive metabolites that deplete ROS scavengers such as glutathione, leading to ROS-induced hepatotoxicity (Figure 3A). Similarly, in the BDL model, ROS serve as a key factor driving hepatocyte damage. Therefore, we first measured ROS generation in the liver tissue. As expected, APAP- or TAA-treated liver tissue exhibited a significant increase in ROS levels, whereas rmSLIT2 treatment effectively suppressed ROS production, restoring levels comparable to those of control groups (Figure 3B). Similarly, APAP-treated cells showed increased ROS generation, while recombinant human SLIT2 (rhSLIT2) protein downregulated its production (Figure 3C). To elucidate the mechanism underlying rmSLIT2-mediated ROS reduction, we performed RNA-seq analysis (Figures S4A–S4C), revealing a downregulation of ROS-related pathways in SLIT2-treated samples (Figure S5A). APAP is metabolized into non-reactive metabolites through sulfation and glucuronidation pathways (Figure 3A). However, rmSLIT2 treatment did not show any significant changes in the expression levels of genes associated with sulfation and glucuronidation (Figure S5B). Additionally, rmSLIT2 treatment (in both the presence and absence of APAP) did not significantly upregulate anti-oxidant proteins compared to controls, suggesting that its protective effects are not mediated by enhanced detoxification pathways or anti-oxidant response (Figure S5C). Therefore, the protective effects of rmSLIT2 treatment were not due to an increase in the conversion of APAP to non-toxic metabolites or a reduction in ROS generation through upregulation of anti-oxidant proteins.

Next, we examined the expression levels of CYP enzymes, a well-known enzyme system involved in drug metabolism and ROS generation.^3^ Consistent with previous findings, APAP treatment led to a significant increase in the expression of CYP enzymes, particularly *Cyp2e1*, which plays a key role in the conversion of APAP into the toxic metabolite *N*-acetyl-*p*-benzoquinone imine. ROS generation was significantly reduced either by treatment with the CYP2E1 inhibitor diallyl sulfide (DAS)^23^^,^^24^ or by siRNA-mediated knockdown of Cyp2e1 in AML12 cells (Figures S6A and S6C). Consistently, DAS co-treatment attenuated APAP-induced CYP2E1 protein upregulation (Figure S6B), and Cyp2e1 knockdown was confirmed at both the mRNA and protein levels (Figures S6D and S6E). These findings demonstrate that increased CYP2E1 expression is a major contributor to ROS-mediated damage in the APAP-induced liver injury model. Notably, rmSLIT2 treatment significantly reduced *Cyp2e1* expression (Figure 3D). To validate these findings, we analyzed RNA-seq data and confirmed the *Cyp2e1* expression pattern using real-time PCR in additional mouse liver tissue samples. Liver tissue treated with APAP or TAA showed a significant increase in CYP2E1 at both the mRNA and protein levels. However, in mice treated with rmSLIT2 in combination with APAP or TAA, CYP2E1 expression was markedly reduced (Figures 3E and 3F). These observations were further confirmed in an *in vitro* cell line model, where APAP treatment increased CYP2E1 expression, while rhSLIT2 treatment effectively suppressed it (Figure S7). A similar pattern was observed in liver tissue from the BDL-induced liver injury model, further supporting the consistent downregulation of Cyp2e1 by SLIT2 (Figures S8A and S8B). To strengthen the causal relationship between SLIT2-mediated CYP2E1 downregulation and reduced ROS generation, CYP2E1 was overexpressed in AML12 cells (Figure 3G), and ROS levels were then measured after APAP treatment to determine whether the effect of SLIT2 would be diminished or abolished. As expected, AML12 cells overexpressing CYP2E1 showed no statistically significant reduction in APAP-induced ROS even after rmSLIT2 treatment (Figure 3H). Taken together, these findings suggest that SLIT2 mitigates ROS generation in toxic liver injury by downregulating Cyp2e1 expression, thereby reducing oxidative stress and limiting hepatotoxicity.

### SLIT2 reduces inflammatory process and CYP2E1 expression in toxic liver injury by regulating the NF-κB pathway

During APAP-induced liver injury, the toxic redox metabolism of APAP produced by CYP2E1 is closely linked to inflammation. Hepatocyte necrosis initiates an inflammatory response, and the amplification of this inflammation can exacerbate liver injury. Since our previous study demonstrated that SLIT2 possesses strong anti-inflammatory properties in aged mice, we aimed to determine whether SLIT2 treatment could alleviate the inflammatory processes associated with APAP-induced liver injury.

**Figure 4:**
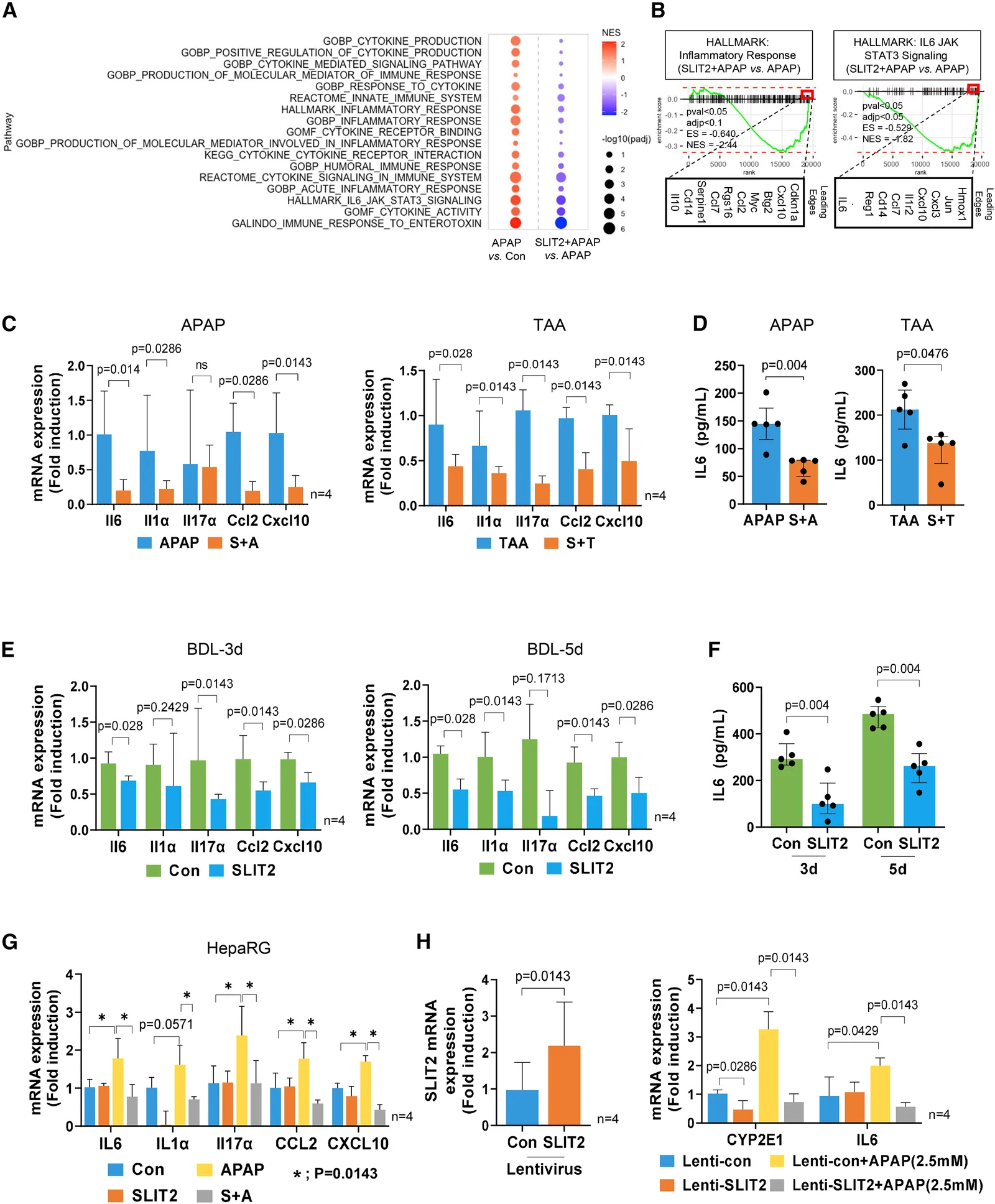
SLIT2 inhibits NF-κB signaling that results in downregulation of CYP2E1 and inflammation (A) Gene set enrichment analysis (GSEA) of inflammation-associated gene sets. GSEA was performed to compare inflammation-related pathways between control vs. APAP-treated (left) and APAP-treated vs. rmSLIT2 + APAP-treated (S + A) (right) liver samples. Enrichment plots illustrate the running enrichment score (ES) across the ranked gene list. (B) Selected Hallmark pathways from GSEA. Statistically significant pathways and enrichment metrics in APAP-treated vs. rmSLIT2 + APAP-treated mouse liver samples are displayed. (C) mRNA expression of key inflammation-related genes in APAP-, TAA-, rmSLIT2 + APAP (S + A)-, and rmSLIT2 + TAA (S + T)-treated mice. (D and F) IL-6 concentrations in serum (*n* = 5 per group). (E) Real-time PCR analysis of inflammation-related genes in liver tissues from BDL mice. (G) Real-time PCR analysis of inflammation-associated genes in HepaRG cells after 24-h treatment under four conditions: control (Con), APAP alone (5 mM), rhSLIT2 alone (200 ng/mL), and combined APAP + rhSLIT2 (S + A). (H) Lentivirus-mediated SLIT2 overexpression in HepaRG cells. Left: SLIT2 expression levels 48 h after transduction with lenti-SLIT2. Right: mRNA expression levels of *CYP2E1* and *IL6* in lenti-SLIT2–transduced HepaRG cells following treatment with or without APAP (2.5 mM) for 24 h, as assessed by quantitative real-time PCR. Real-time PCR experiments were performed in four independent replicates. Data are presented as median ± IQR, and statistical significance was determined using the Mann-Whitney U test.

**Figure 5:**
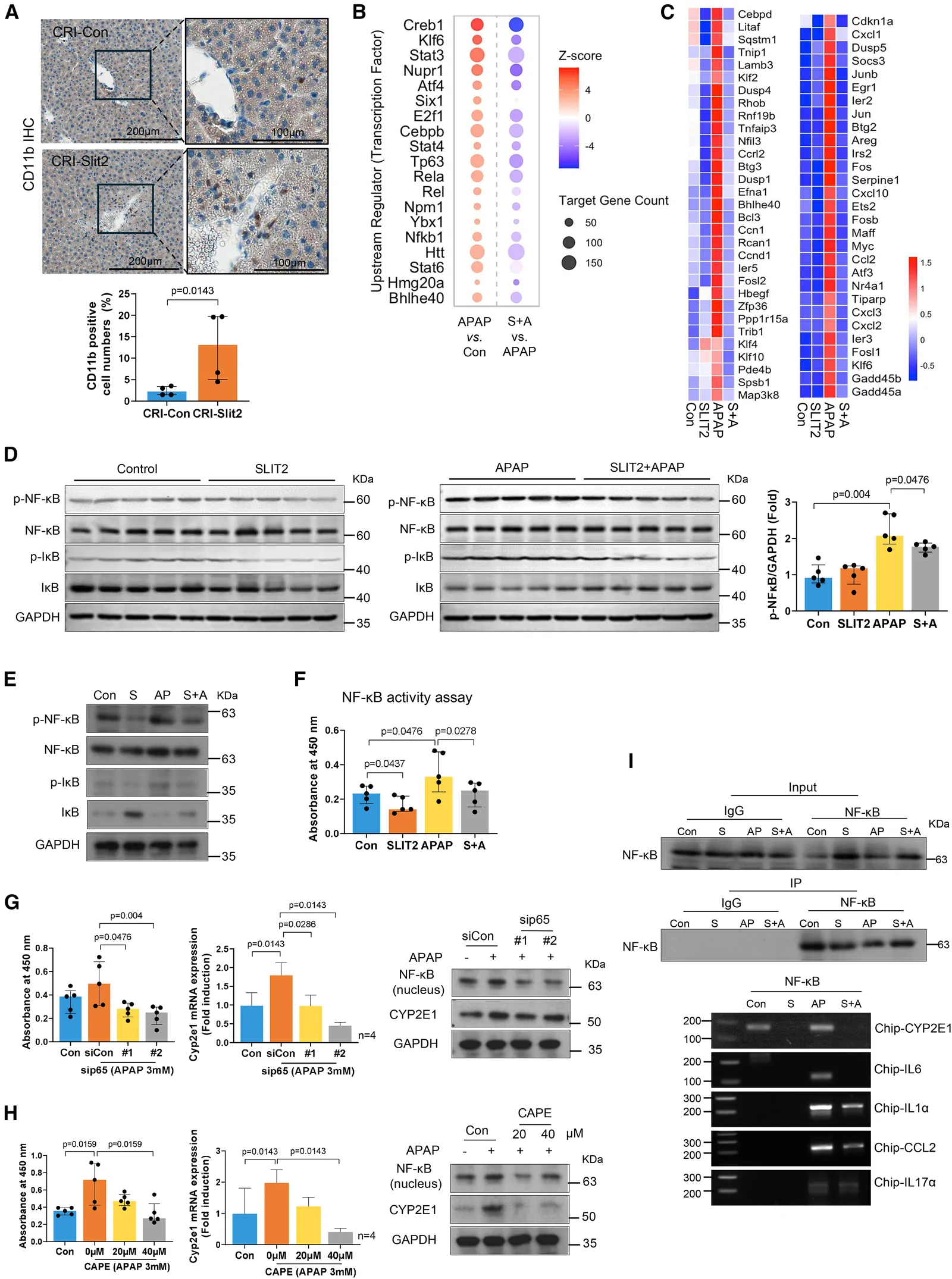
SLIT2 inhibits NF-κB signaling and transcriptional regulation of inflammatory and oxidative stress genes (A) IHC analysis of CD11b. Representative CD11b-stained liver tissue images from CRI-Con and CRI-Slit2 groups are shown. The lower panel presents quantification of CD11b-positive cells per field (*n* = 4). (B) Ingenuity Pathway Analysis of upstream regulators from RNA-seq data. Predicted upstream regulators were identified in APAP-treated vs. control (left) and rmSLIT2 + APAP-treated vs. APAP-treated (right) liver samples, highlighting key transcription factors involved in APAP-induced hepatotoxicity and SLIT2-mediated modulation. (C) Heatmap of genes associated with the HALLMARK: TNFA signaling via NF-κB pathway, showing expression patterns across different treatment groups. (D) Western blot analysis of NF-κB signaling in APAP-treated liver tissues. Protein levels of total NF-κB, phosphorylated NF-κB (p-NF-κB), IκB, phosphorylated IκB (p-IκB), and GAPDH were analyzed. The right panel shows quantification of p-NF-κB levels relative to GAPDH. (E) Western blot analysis of NF-κB pathway components in HepaRG cells. Protein levels of p-NF-κB, IκB, p-IκB, and GAPDH (loading control) were assessed. (F) NF-κB activity was assessed in HepaRG cells by isolating nuclear extracts and measuring NF-κB nuclear translocation. (G) HepaRG cells were transfected with siControl or sip65 for 48 h, followed by APAP (5 mM) treatment for 24 h. Left: NF-κB activity was measured in nuclear extracts. Middle: *CYP2E1* mRNA expression was quantified by real-time PCR. Right: CYP2E1 protein levels were analyzed by western blot. sip65 transfection suppressed CYP2E1 protein expression, indicating that NF-κB activity contributes to CYP2E1 induction. GAPDH served as a loading control. (H) HepaRG cells were treated for 24 h under four conditions: control (Con), APAP (5 mM), CAPE (20 or 40 μM), or a combination of APAP and CAPE. Left: NF-κB activity assay showing nuclear translocation. CAPE significantly attenuated APAP-induced NF-κB activation. Middle: quantitative PCR of *CYP2E1* mRNA expression. Right: western blot analysis of CYP2E1 protein levels confirmed the suppression by CAPE. GAPDH was used as the loading control. (I) ChIP assay in HepaRG cells to assess NF-κB binding to the promoter regions of *CYP2E1*, *IL6*, *IL1α*, *CCL2*, and *IL17α*. The upper panel shows input DNA as a loading control; the lower panel shows PCR results for NF-κB-immunoprecipitated DNA. For (E), (F), and (I), cells were treated for 24 h under four conditions: control (Con), APAP alone (5 mM), rhSLIT2 alone (200 ng/mL), and combined APAP + rhSLIT2 (S + A). Real-time PCR experiments were performed in four independent replicates. Data are presented as median ± IQR, and statistical significance was determined using the Mann-Whitney U test.

Gene sets associated with inflammatory responses and related signaling pathways were significantly enriched in APAP-treated liver samples, whereas rmSLIT2 co-treatment resulted in a marked reduction in the enrichment of these gene sets (Figures 4A and 4B). Real-time PCR and serum ELISA analysis of interleukin-6 (IL-6) also showed downregulation of inflammation-related gene expression following rmSLIT2 treatment in both APAP- and TAA-induced liver injury models (Figures 4C and 4D). Moreover, elevated mRNA expression levels of several key inflammatory cytokines and chemokines were also observed in the BDL-induced liver injury model, but these were significantly downregulated following rmSLIT2 treatment (Figures 4E and 4F). We further validated these findings in the HepaRG cell line. APAP treatment increased mRNA expression of inflammatory cytokines and chemokines, while rhSLIT2 treatment effectively downregulated their expression (Figure 4G). Additionally, SLIT2 overexpression via lentivirus also reduced *Cyp2e1* and *Il6* expression in APAP-treated hepatocytes (Figure 4H). Furthermore, liver-specific knockdown of *Slit2* in mice led to a significant increase in inflammatory cell infiltration compared to controls (Figure 5A). These results collectively suggest that SLIT2 mitigates liver damage by attenuating inflammatory responses in hepatic toxicity models.

To identify the key transcriptional regulators involved in the suppression of CYP2E1 and inflammatory genes by SLIT2, we conducted Ingenuity Pathway Analysis (IPA) using RNA-seq data. As shown in Figure 5B, several upstream transcription factors, including Creb1, Klf6, Stat3, and Rela, showed substantial changes. Interestingly, most of the regulators from Creb1 to Rela are known to be associated with the NF-κB signaling pathway. These findings suggest that the regulatory effects of SLIT2 are closely linked to modulation of NF-κB-related transcriptional activity. Consistently, heatmap analysis of NF-κB signaling genes showed that APAP-induced activation of tumor necrosis factor α (TNF-α)/NF-κB signaling was significantly attenuated by SLIT2 (Figure 5C). This was confirmed by western blot analysis of liver tissues showing reduced levels of phosphorylated NF-κB in the SLIT2-treated group (Figure 5D). *In vitro* validation using HepaRG cells showed that APAP exposure enhanced NF-κB phosphorylation, which was suppressed by rhSLIT2 co-treatment (Figure 5E). Furthermore, SLIT2 treatment significantly reduced NF-κB nuclear translocation, as evidenced by decreased activity in nuclear extracts (Figure 5F). To determine whether CYP2E1 expression is regulated by NF-κB, we performed experiments using NF-κB inhibition. Notably, knockdown of the NF-κB subunit p65 (siP65) reduced p65 levels in nuclear extracts (Figure 5G, left) and abolished the SLIT2-induced suppression of CYP2E1 protein expression, suggesting that NF-κB plays a key intermediary role in this regulatory pathway (Figure 5G, middle and right). Consistently, similar results were observed when NF-κB was suppressed using the NF-κB inhibitor caffeic acid phenethyl ester (CAPE) (Figure 5H).

We next performed chromatin immunoprecipitation (ChIP) assays to determine whether NF-κB directly binds to the promoter regions of its target genes. Using the University of California, Santa Cruz (UCSC) Genome Browser, we identified NF-κB consensus binding motifs (GGGRNNYYCC) in the promoter regions of each gene. For each target, we selected one representative site and designed PCR primers covering 2–3 candidate regions. ChIP-PCR analysis confirmed NF-κB binding at the promoters of CYP2E1, IL-6, IL-1α, CCL2, and IL-17α. Notably, SLIT2 treatment significantly reduced NF-κB occupancy at these promoter regions, confirming that SLIT2 modulates transcriptional activity via NF-κB inhibition (Figure 5I). These findings support a model in which SLIT2 attenuates hepatic inflammation and oxidative stress by suppressing NF-κB signaling and its downstream transcriptional targets, including CYP2E1.

### ROBO4 mediates the hepatoprotective effects of SLIT2

**Figure 6:**
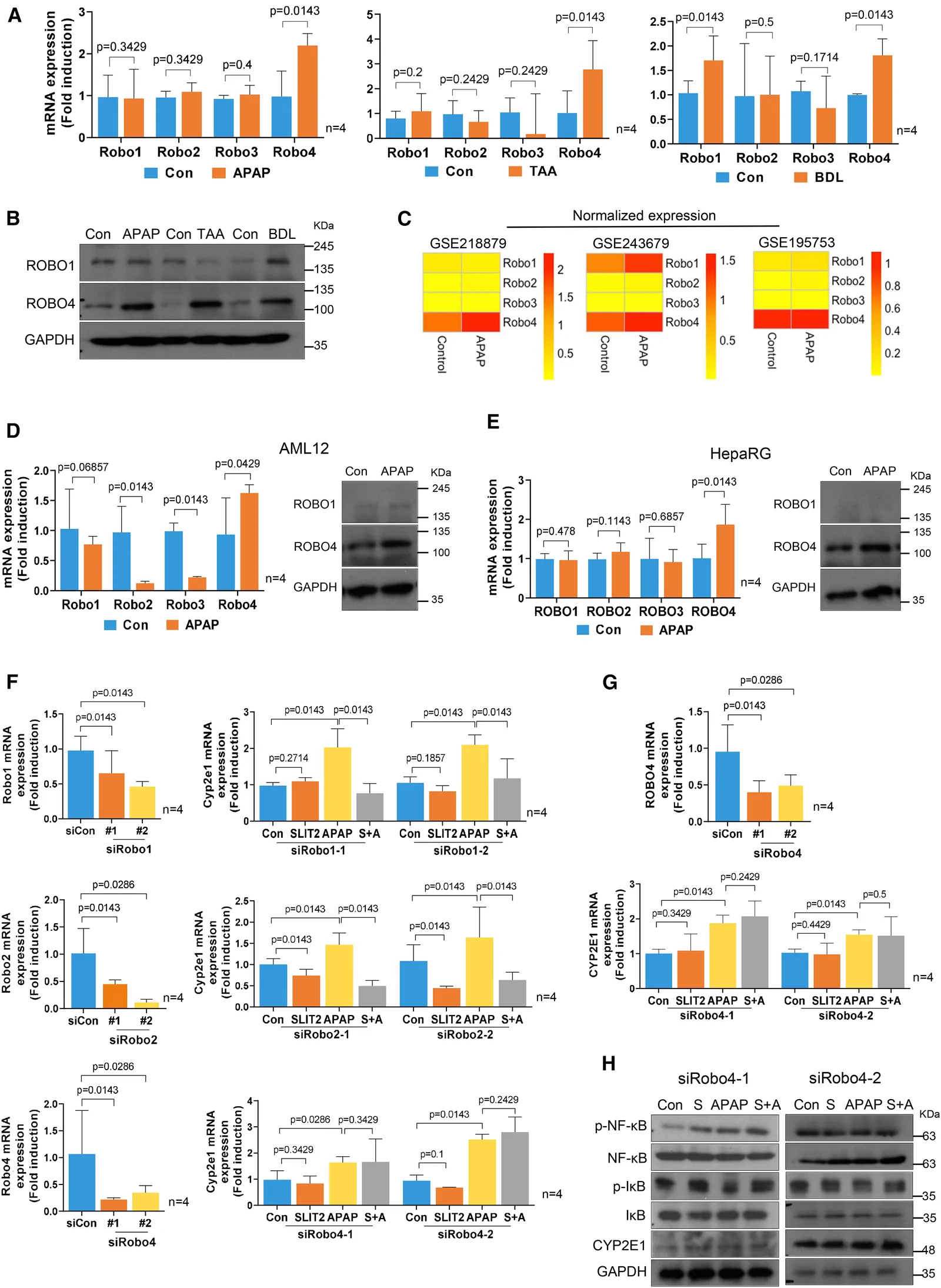
ROBO4 is a key mediator of SLIT2-dependent hepatoprotection pathways (A) mRNA expression levels of *Robo1–4* in APAP-treated, TAA-treated, and BDL mouse liver. (B) Protein expression levels of ROBO1 and ROBO4 in APAP-treated, TAA-treated, and BDL mouse liver. (C) Normalized expression levels of *Robo1–4* based on publicly available RNA-seq datasets from control and APAP-treated mouse liver. (D) mRNA and protein expression levels of *Robo1–4* in AML12 cells following 3 mM APAP treatment for 24 h. Expression was analyzed by real-time PCR (left) and western blotting for ROBO1 and ROBO4 (right). (E) mRNA and protein expression levels of *Robo1–4* in HepaRG cells after 5 mM APAP treatment for 24 h. Left: real-time PCR. Right: western blot analysis of ROBO1 and ROBO4. (F) Knockdown of *Robo1*, *Robo2*, and *Robo4* in AML12 cells using siRNA. Left: expression levels of each gene following si#1 and si#2 transfection. Right: *Cyp2e1* expression under each knockdown condition, analyzed by real-time PCR. (G) *Robo4* expression in HepaRG cells following siRobo4 transfection (upper) and *Cyp2e1* expression after rhSLIT2 or APAP treatment (lower) assessed by real-time PCR. Cells were treated for 24 h under four conditions: control (Con), APAP alone (5 mM), rhSLIT2 alone (200 ng/mL), and combined APAP + rhSLIT2 (S + A). (H) Western blot analysis of p-NF-κB, p-IκB, and CYP2E1 expression in AML12 cells following siRobo4-1 and siRobo4-2 transfection. In (F) and (H), AML12 cells were treated for 24 h under the same four conditions: control (Con), APAP alone (3 mM), rmSLIT2 alone (200 ng/mL), and combined APAP + rmSLIT2 (S + A). Real-time PCR experiments were performed in four independent replicates. S + A indicates the SLIT2 + APAP-treated group. For Real-time PCR, data are presented as median ± IQR, and statistical significance was determined using the Mann-Whitney U test.

SLIT2 can bind to ROBO receptors (ROBO1–4), and its function may vary depending on the ROBO receptor subtype. To identify which ROBO interaction with SLIT2 confers hepatoprotective effects, we first examined ROBO receptor expression in liver injury models. The results showed that ROBO4 mRNA and protein levels were consistently increased, whereas the expression of ROBO1–3 either remained unchanged or was reduced following liver injury induced by APAP, TAA, and BDL, except for elevated ROBO1 expression after BDL (Figures 6A and 6B). IHC further demonstrated a significant increase in ROBO4 expression (Figure S9A) but not ROBO1 expression (Figure S9B) in damaged liver tissue from all three mouse models. When E-cadherin was used as a marker for hepatocyte membranes, ROBO4 was predominantly localized to the hepatocyte membrane and closely paralleled the E-cadherin staining pattern, with additional staining observed in certain non-parenchymal cell populations in the BDL model. Additionally, analysis of public RNA-seq datasets (GEO: GSE218879, GSE243679, and GSE195753) from APAP-induced liver injury mouse models confirmed a consistent upregulation of *Robo4* among all ROBO receptor families (Figure 6C). *Robo4* expression was increased by APAP-treated cells (Figure 6D). Consistent with findings in the mouse hepatocyte cell line, HepaRG, a human hepatocyte cell line, also exhibited ROBO4 upregulation after APAP treatment (Figure 6E). To elucidate the hepatoprotective role of the SLIT2/ROBO4 interaction, we generated knockdowns of *Robo1*, *Robo2*, and *Robo4* in AML12, a mouse hepatocyte cell line, using two different siRNAs for each receptor subtype. Interestingly, *Robo4* downregulation completely abrogated the hepatoprotective effects of rmSLIT2 treatment, including the reduction of *Cyp2E1* expression during APAP treatment (Figure 6F). This finding, along with the lack of change in *Cyp2E1* expression following *Robo4* knockdown, further supports the role of ROBO4 in regulating CYP2E1 (Figure 6G). Furthermore, when *Robo4* expression was knocked down by siRNA, the increased phosphorylation of NF-κB following APAP treatment was not suppressed by rmSLIT2 treatment (Figure 6H). Collectively, these findings suggest that SLIT2/ROBO4 signaling is upregulated in response to toxic substance exposure and may play a critical role in mitigating hepatotoxicity.

### Development of SLIT2-derived peptides for mitigating hepatotoxicity

**Figure 7:**
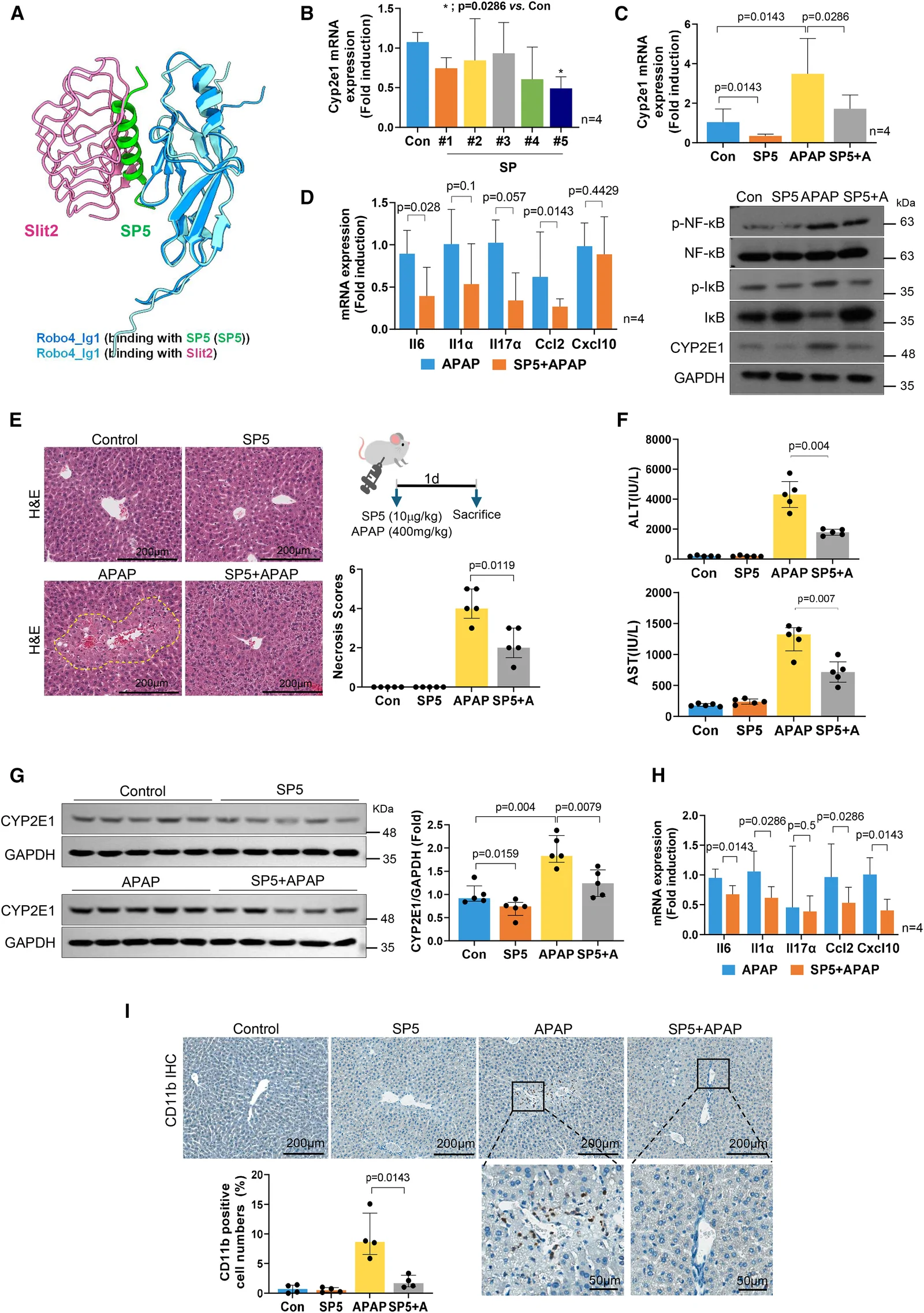
SLIT2-derived peptide 5 suppresses inflammation and CYP2E1 expression via ROBO4 (A) Structural model of the SLIT2-ROBO4 complex and SLIT2-derived peptide 5 (SP5) interaction. The predicted structures of the mouse SLIT2 LRR domain (pink) bound to the ROBO4 Ig1 domain (light blue), and the SP5 peptide (green) bound to the ROBO4 Ig1 domain (blue), were generated using AlphaFold3 software. (B) *Cyp2e1* mRNA expression in AML12 cells treated with SLIT2-derived peptides SP1–SP5. Cells were treated with each peptide for 24 h, and *Cyp2e1* expression was analyzed using real-time PCR. (C and D) (C) AML12 cells were treated with SP5 alone or in combination with APAP. *Cyp2e1* mRNA expression was assessed by real-time PCR. (D, left) Expression of inflammation-related genes in AML12 cells following SP5 and/or APAP treatment. (D, right) Western blot analysis of p-NF-κB, p-IκB, and CYP2E1 protein levels under the same treatment conditions. (E) SP5 protects against APAP-induced liver injury *in vivo*. Mice were treated simultaneously with SP5 (10 μg/kg) and APAP (400 mg/kg). H&E staining was used to evaluate liver histopathology; the right panel shows necrosis scores. (F) Serum ALT and AST levels in SP5-treated and/or APAP-treated mice. (G) Western blot analysis of CYP2E1 protein expression in liver tissues from SP5-treated and/or APAP-treated mice. Protein bands were quantified using densitometry, with expression levels normalized to GAPDH. (H) Real-time PCR analysis of inflammation-related gene expression in AML12 cells treated with APAP or APAP + SP5. (I) IHC analysis of CD11b-positive immune cell infiltration in liver tissues from mice treated with SP5 and/or APAP. Real-time PCR experiments were performed in four independent replicates. In (C) and (D), AML12 cells were treated for 24 h under four conditions: control (Con), APAP alone (3 mM), SP5 alone (100 ng/mL), and combined APAP + SP5 (SP5+A). Data are presented as median ± IQR, and statistical significance was determined using the Mann-Whitney U test.

We aim to develop SLIT2 as a hepatoprotective agent. However, the large size of SLIT2 protein poses a challenge for its direct application in mitigating hepatocyte toxicity. Moreover, to date, no ROBO4 receptor agonists have been developed. To overcome these limitations, we designed smaller peptides capable of activating the ROBO4 receptor. These peptides were derived from the LRR2 domain of SLIT2, a region critical for receptor binding. To enhance stability and functionality, the peptides were synthesized with alanine residues linked to their sequence (Figure 7A). Among the five designed SLIT2-derived peptides (#1–5), SP5 exhibited significantly greater suppression of *Cyp2E1* compared to the other peptides (Figure 7B) and, by binding to the immunoglobulin 1 (Ig1) domain, demonstrated activity comparable to that of the full-length SLIT2 protein. Peptide docking simulation scores between the Ig1 and Ig2 domains of ROBO1–4 and SP5 predicted that SP5 binds most strongly to the Ig1 domain of ROBO4, further supporting the possibility that SP5 may exhibit the highest affinity for ROBO4 among the four receptors under physiological conditions (Table S1). Based on this specificity, we evaluated the efficacy of the SP5 in AML12 cells following APAP treatment. SP5 treatment significantly reduced the expression of *Cyp2E1* (Figure 7C), a key enzyme involved in toxic substance metabolism, as well as the expression of pro-inflammatory genes, accompanied by a reduction in NF-κB activation (Figure 7D). Notably, these hepatoprotective effects were abolished upon *Robo4* knockdown using siRNA (Figure S10A) but remained unaffected by the knockdown of *Robo1* or *Robo2* (Figure S10B). Furthermore, in an APAP-induced liver injury mouse model, SP5 markedly reduced liver necrosis as well as serum AST and ALT levels (Figures 7E and 7F) and CYP2E1 protein expression following APAP treatment (Figure 7G). Additionally, inflammation-related gene expression and inflammatory cell infiltration, assessed by CD11b expression, was significantly reduced by SP5 treatment (Figures 7H and 7I). Taken together, these findings suggest that the SLIT2-derived peptide is a promising therapeutic candidate for mitigating liver toxicity through ROBO4 signaling activation.

### Post-treatment with SLIT2 and SP5 attenuates APAP-induced hepatotoxicity and inflammation

**Figure 8:**
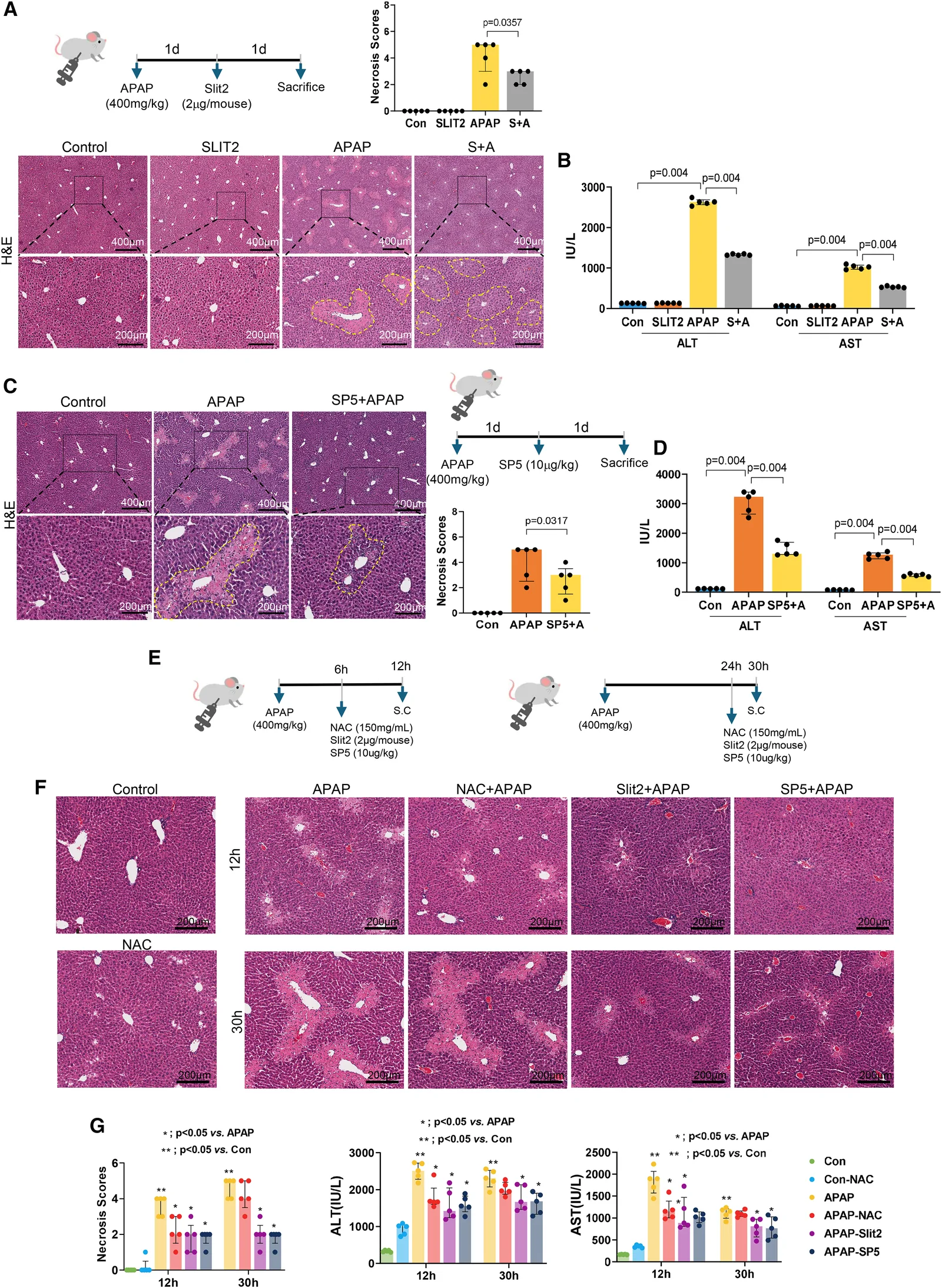
Post-treatment with SLIT2 or SP5 peptide alleviates APAP-induced liver injury (A) Experimental scheme for rmSLIT2 post-treatment. Mice received APAP (400 mg/kg, i.p.), followed 24 h later by recombinant SLIT2 (rmSLIT2; 2 μg/mouse, subcutaneous). Mice were sacrificed 24 h after rmSLIT2 administration (48 h after APAP). (B) Serum ALT and AST levels measured 48 h after APAP administration. (C) Experimental design for SP5 post-treatment. Mice were treated with SP5 (10 μg/kg) 24 h after APAP injection, followed by tissue and serum analysis 24 h later. Representative H&E-stained liver sections and quantification of necrotic areas. (D) Serum ALT and AST levels in SP5-treated groups (*n* = 5 per group). (E) Experimental scheme. Mice were fasted for 12 h and administered APAP (400 mg/kg, oral). At 6 h or 24 h after APAP, mice received recombinant SLIT2 (2 μg/mouse, subcutaneous), SP5 peptide (10 μg/kg, subcutaneous), or NAC (300 mg/kg, i.p.). All other groups were sacrificed 6 h after each post-treatment. Control groups received PBS (Con) or PBS plus NAC (Con-NAC) at 0 h and were sacrificed at 30 h. (F) Representative H&E-stained liver sections. APAP induced severe centrilobular necrosis. NAC was protective only at 6 h post APAP, whereas SLIT2 and SP5 reduced necrosis even at 24 h. (G) Quantification of liver injury. Left: necrosis scores (0–5 scale). Middle and right: serum ALT and AST levels. Data are presented as median ± IQR, and statistical significance was determined using the Mann-Whitney U test.

To evaluate the therapeutic potential of SLIT2 and its derived peptide SP5 in a clinically relevant post-injury setting, we employed a delayed treatment model in which APAP was administered first, followed by SLIT2 or SP5 administration 24 h later. Mice were then sacrificed 24 h after rmSLIT2 treatment (Figure 8A, upper left). Histological analysis of liver tissue revealed that post-treatment with SLIT2 significantly reduced hepatic necrosis compared to APAP treatment alone (Figure 8A, lower). Quantification of necrotic areas confirmed a marked reduction in necrosis scores in the rmSLIT2-treated group compared to the APAP-only group (Figure 8A, upper right). In parallel, serum levels of ALT and AST, indicators of liver injury, were significantly decreased following rmSLIT2 post-treatment (Figure 8B).

To further investigate the efficacy of SP5 in this delayed intervention setting, mice received SP5 24 h after APAP injection. Similar to rmSLIT2, SP5 post-treatment substantially ameliorated histological liver damage and significantly reduced serum transaminase levels (Figures 8C and 8D). Mechanistically, SP5 reduced hepatic CYP2E1 protein expression (Figure S11A) and suppressed mRNA levels of inflammatory mediators including *Il6*, *Il1β*, and *Ccl2* (Figure S11B). Moreover, immunohistochemical staining for CD11b revealed a notable reduction in immune cell infiltration in the SP5-treated livers, consistent with dampened inflammatory responses (Figure S11C). Finally, the efficacy of rmSLIT2, SP5, and NAC was compared at early (6 h) and late (24 h) treatment time points following APAP injection (Figures 8E and 8F). Consistent with previous studies, NAC showed protective effects against APAP-induced liver damage when administered at the early time point but not at the late time point. The effects of rmSLIT2 and SP5, as demonstrated by histological improvement and reduced serum liver enzyme levels, were comparable to those of NAC at the early time point. Furthermore, we clearly observed superior hepatoprotective effects of rmSLIT2 and SP5 compared to NAC even when treatment was initiated 24 h after APAP insult (Figure 8G). These results clearly demonstrate that both SLIT2 and its functional peptide SP5 retain hepatoprotective efficacy even when administered after the onset of liver injury, highlighting their potential as therapeutic agents for treating established toxic liver damage.

## Discussion

Drug-induced liver injury accounts for approximately one-tenth of acute hepatitis cases and up to half of acute liver failure cases in western countries.^25^ However, the primary treatment for drug-induced liver injury remains largely limited to the withdrawal of the causative drug, except for the use of NAC in APAP toxicity and L-carnitine supplementation in cases of valproic acid overdose.^26^ Moreover, NAC is effective only at the early phase (<6 h) of APAP-induced acute liver injury.^27^ Notably, in patients with non-APAP-induced acute liver injury, a meta-analysis of 11 studies demonstrated that NAC reduced mortality and the incidence of hepatic encephalopathy compared to standard care.^8^ However, developing effective and reliable medications to prevent the progression of acute hepatitis to acute liver failure remains a significant clinical unmet need. The discovery of natural proteins or associated signaling pathways that protect liver parenchyma during toxic injury could serve as an important starting point. For example, C-reactive protein (CRP), a commonly induced inflammatory marker produced by hepatocytes, was recently identified as a crucial checkpoint that limits the destructive activation of complement, which can amplify inflammation in the late phase of APAP-induced acute liver injury. Therefore, unlike NAC, CRP treatment has been shown to be highly effective even beyond 6 h after the onset of liver injury.^28^ In the present study, among the ligands in the SLIT/ROBO pathway, SLIT2 was predominantly upregulated in liver tissue and serum from mouse models of liver injury as well as in the serum of a patient cohort with toxic hepatitis of various etiology. Treatment with recombinant SLIT2 protein or its peptide conferred protection against toxic liver injury by reducing NF-κB activity, thereby suppressing the transcription of CYP2E1 and several inflammatory cytokines. Moreover, similar to CRP treatment,^28^ administration of SLIT2—even up to 24 h after toxic insult—still exerted significant hepatoprotective effects. These findings suggest that SLIT2-based therapy may offer greater clinical applicability than NAC, particularly in real-world scenarios where early intervention such as within 6 h is not always possible.

Previous studies have suggested that SLIT2 exhibits tissue-protective functions in various injury models, including neuroinflammation during germinal matrix hemorrhage,^15^ surgical brain injury,^16^ kidney ischemia-reperfusion injury and associated fibrosis,^21^ heart ischemia-reperfusion injury,^29^ and congestive heart disease.^18^ Although the underlying molecular mechanisms remain to be fully elucidated, the anti-inflammatory effects of SLIT2 have been consistently observed across these tissue injury models as well as in the liver injury examined in the present study. The anti-inflammatory effects of SLIT2 can be partially explained by its inhibition of immune cell chemotaxis,^30^ which occurs through the suppression of chemoattractant-induced activation of Rho guanosine triphosphatases (Rac2 and Cdc42)^31^ or the tyrosine phosphorylation of focal adhesion components (RAFTK/Pyk2, focal adhesion kinase, and paxillin) via Src family kinases.^32^ In addition to its role in immune cell chemotaxis, SLIT2 also regulates inflammatory cytokine production, a key process in controlling the inflammatory response. Notably, NF-κB signaling, a well-established mediator of pro-inflammatory gene induction,^33^ has been shown to be negatively regulated by SLIT2 through the inhibition of LPS-induced phosphorylation of RAFTK/Pyk2.^19^ In the liver injury models examined in this study, increased NF-κB signaling activity was closely associated with prolonged inflammation and exacerbated tissue damage.^34^ Although the precise molecular mechanism by which SLIT2-bound ROBO receptors—despite lacking intrinsic catalytic or enzymatic activity—suppress NF-κB transcriptional activity remains to be elucidated, treatment with recombinant SLIT2 protein or its peptide significantly reduced injury-induced NF-κB activity not only in *in vivo* mouse liver tissues but also in *in vitro* human and mouse liver cell lines. The modulation of NF-κB activity by SLIT2 treatment influences the expression of several pro-inflammatory genes as well as the regulation of CYP2E1 levels. In addition to NF-κB, several transcription factors, including β-catenin, HNF-1, specificity protein 1 (SP1), TonEBP/NFAT5, STAT6, NFATc1, and STAT3, have been implicated in the transcriptional regulation of Cyp2e1 in various cell types. It has been reported that pro-inflammatory cytokines such as IL-6, IL-1α, IL-1β, and TNF-α can directly activate these transcription factors. For example, IL-6 increases CYP2E1 expression in colorectal cancer cell lines via a transcriptional mechanism involving STAT3.^35^ IL-1α treatment induces SP1 binding to the CYP2E1 promoter in human hepatoma cells.^36^ IL-1β and TNF-α enhance the expression and nuclear translocation of TonEBP/NFAT5 in fibroblast-like synoviocytes.^37^ In addition, NF-κB can directly control the induction of NFATc1.^38^^,^^39^ Therefore, unlike NAC, which is primarily effective during the early phase of APAP-induced toxic liver injury and acts mainly by replenishing glutathione to counteract ROS damage, SLIT2 treatment may confer sustained hepatoprotective effects throughout the entire course of toxic liver injury.

Among the ROBO receptors, ROBO1–3 consist of five Ig-like domains and three fibronectin type III repeats, whereas ROBO4 contains only two Ig-like domains and one fibronectin type III domain.^40^ Unlike ROBO1–3, the transcriptional regulation of ROBO4 has been more extensively studied. Its basal promoter activity is regulated by GA-binding protein^41^ and SP1,^42^ and the activation of SP1 and NF-κB has been shown to upregulate ROBO4 expression under conditions of hyperglycemia^43^ and inflammation.^44^ These findings are consistent with our observation that, among the ROBO receptors, the mRNA and protein levels of ROBO4 were consistently elevated in liver injury models induced by APAP treatment, TAA treatment, and BDL. Previous studies suggest that the function of ROBO4 is specific to the vasculature.^45^ However, public databases, including the Human Protein Atlas and transcriptomics datasets (HPA and GTEx), indicate that ROBO4 is expressed in seven liver cell types: hepatocytes, vascular endothelial cells, fibroblasts, smooth muscle vessel cells, T cells, plasma cells, B cells, and macrophages.^40^ Therefore, beyond its role in vascular endothelial cells, further studies are needed to elucidate the function of ROBO4 upregulation in various liver cell types during inflammation associated with liver disease. Interestingly, our findings strongly suggest that the liver-protective effects of SLIT2 may be primarily dependent on ROBO4 expressed in hepatocytes. The comparable protective effects observed between full-length recombinant SLIT2 and the ROBO4-specific SLIT2 peptide further support the importance of the SLIT2/ROBO4 interaction in toxic liver injury.

Despite this study providing strong evidence supporting the hepatoprotective effect of SLIT2 in acute and subacute liver injury, these findings appear somewhat contradictory to previous reports that described the pro-fibrotic activity of SLIT2 in a liver cirrhosis model using SLIT2 transgenic mice. Nonetheless, since SLIT2 has been shown to attenuate fibrosis in kidney and cardiac injury models, it is more plausible that SLIT2 is not directly involved in fibrogenesis itself but rather modulates tissue repair and fibrosis in a context-dependent manner. For example, hepatocyte-specific STAT3 knockout mice develop more severe liver fibrosis compared with wild-type mice across various experimental fibrosis models, suggesting a hepatoprotective role for STAT3,^46^ while leptin, a well-established pro-fibrotic mediator, induces STAT3 activation in hepatic stellate cells (HSCs), and inhibition of STAT3 signaling reduces leptin-mediated HSC survival and activation.^47^ Moreover, STAT3 contributes to leptin-induced production of TIMP-1, a key survival factor for HSCs.^48^ Collectively, these findings suggest that STAT3 protects against hepatocyte damage, thereby reducing injury-driven liver fibrosis while promoting HSC survival and proliferation followed by enhanced liver fibrosis.^49^ Similarly to STAT3 activation, although further studies are warranted, we cautiously propose that SLIT2 protects hepatocytes from acute injury primarily via ROBO4 receptor signaling, whereas in chronic liver injury, SLIT2 may promote HSC activation thorough ROBO1 receptor signaling.

Although numerous preclinical studies have demonstrated the beneficial clinical potential of recombinant SLIT2 protein treatment in various disease conditions, only one phase 2 clinical trial has been conducted using a ROBO2 fusion protein (PF-06730512), a neutralizing ligand trap that disrupts SLIT2/ROBO2 interactions, in patients with focal segmental glomerulosclerosis.^50^ The limited number of clinical trials investigating SLIT/ROBO signaling may be attributed to the absence of more detailed studies identifying the key receptor-ligand pairs that function as primary effectors in specific disease contexts, as well as the pleiotropic effects of SLIT2, which vary depending on the ROBO receptor subtype even within the same disease condition. In light of these challenges, our SLIT2 peptide may offer greater therapeutic applicability compared to full-length recombinant SLIT2, not only in toxic liver injury but also in other liver inflammatory diseases characterized by ROBO4 upregulation. In addition, while SLIT2 has been reported to exhibit both pro-tumorigenic^29^^,^^30^^,^^31^ and anti-tumorigenic functions,^45^ ROBO4, an effective tumor endothelial marker,^51^ has been shown to suppress breast cancer growth and metastasis by regulating tumor angiogenesis.^52^ Therefore, it would be of great interest to investigate whether our more ROBO4-specific SLIT2 peptide, unlike full-length recombinant SLIT2, could exhibit enhanced anti-tumorigenic activity by more selectively inhibiting angiogenesis.

In conclusion, the present study identifies SLIT2/ROBO4 signaling as a key protective pathway in toxic liver injury. SLIT2 attenuates liver damage by suppressing NF-κB activation and CYP2E1 expression, thereby reducing oxidative stress and inflammation even when administered up to 24 h after toxic insult. A SLIT2-derived peptide that more specifically targets ROBO4 demonstrates efficacy comparable to that of full-length SLIT2. Collectively, these findings support the therapeutic potential of targeting the SLIT2/ROBO4 axis as a promising strategy to prevent fatal fulminant hepatitis in clinically heterogeneous forms of toxic liver injury.

## Materials and methods

### Human serum collection

This study was conducted in accordance with the ethical principles outlined in the Declaration of Helsinki and was approved by the Institutional Review Board (IRB) of Ajou University Hospital (approval no. AJOUIRB-EX-2024-425, Suwon, Korea). The IRB granted an exemption from the requirement for informed consent. Serum samples were collected from a total of 40 individuals, including 20 participants in the normal group and 20 in the toxicity-exposed group. The biospecimens and data used for this study were provided by the Biobank of Ajou University Hospital, a member of Korea Biobank Network. Detailed demographic and clinical information of the participants is provided in Table S2.

### Liver injury animal experiments

Male C57BL/6J mice (6 weeks old) were used in this study, and all experimental procedures were approved by the Institutional Animal Care and Use Committee of Ajou University Medical Center (approval no. 2024-0011). Mice were randomly assigned to experimental groups (*n* = 5 per group) and subjected to different liver injury models. For the APAP-induced liver injury model, mice received intraperitoneal (i.p.) injections (2 μg/mouse) of rmSLIT2 protein (5444-SL; R&D Systems, Minneapolis, MN) or 1× PBS once daily for three consecutive days as a pretreatment. On day 3, a single dose of APAP (400 mg/kg) (103-90-2; Merck, Darmstadt, Germany) or 1× PBS was administered via oral gavage and, after 24 h, mice were sacrificed and biological samples collected. For the TAA-induced liver injury model, mice were treated with TAA (62-55-5; Sigma-Aldrich, St. Louis, MO) for 4 weeks, receiving 50 mg/kg in the first week, 100 mg/kg in the second and third weeks, and 150 mg/kg in the fourth week. TAA was administered intraperitoneally three times per week, and at the time of TAA administration, mice received i.p. injections of either rmSLIT2 (2 μg/mouse) or 1× PBS. After 4 weeks, mice were sacrificed and biological samples collected. For the BDL model, mice were divided into groups based on post-surgical time points (3 days or 5 days). Immediately after surgery, mice received a single i.p. injection of rmSLIT2 (2 μg/mouse) or 1× PBS. The BDL procedure was performed under anesthesia by making a midline abdominal incision, lifting the liver to expose the common bile duct, double-ligating the bile duct with 7.0 surgical silk sutures, and closing the incision layers. For the SP5 experiment, mice received i.p. injections of SP5, followed by a single dose of APAP (400 mg/kg) or 1× PBS via oral gavage on the same day, and after 24 h the animals were sacrificed for sample collection. At the designated time points, mice were euthanized, and blood was collected via intracardiac puncture for serum isolation. Liver tissues were harvested for histological analysis (paraffin-embedded and frozen sections), western blotting, and RNA extraction.

For post-treatment SLIT2 and SP5 experiments, mice received a single i.p. injection of APAP (400 mg/kg). Twenty-four hours later, animals were administered either recombinant SLIT2 (2 μg/mouse, subcutaneous) or SP5 (10 μg/kg, subcutaneous). Control groups received PBS. All mice were sacrificed 24 h after treatment (48 h after APAP administration).

For the NAC experiment, male C57BL/6J mice (6 weeks old) were fasted for 12 h to enhance susceptibility to hepatotoxic injury. A single dose of APAP (400 mg/kg via oral gavage) was administered to induce acute liver injury. To evaluate delayed therapeutic efficacy, mice received i.p. injections of NAC (300 mg/kg) (A9165; Sigma-Aldrich) either 6 h or 24 h after APAP administration. In parallel, other groups were treated subcutaneously with either recombinant SLIT2 protein (2 μg/mouse) or SP5 peptide (10 μg/kg) at the same post-APAP time points. Control groups received PBS both orally and intraperitoneally (Con), or PBS orally and NAC intraperitoneally (Con-NAC). All mice were sacrificed 6 h after the respective post-treatment, and serum and liver tissues were harvested for biochemical assays, histological evaluation, and molecular analyses.

### Knockdown animal experiments

CRISPR-Slit2-AAV8 (CRI-Slit2) was designed and synthesized by VectorBuilder and consists of pAAV[SagRNA]-CAG>mCherry-U6>mSlit2 (GAA AGC CTT CCT TGG AAT TGC GTT TTA GTA CTC TGG AAA CAG AAT CTA CTA AAA CAA GGC AAA ATG CCG TGT TTA TCT CGT CAA CTT GTT GGC GAG A) along with pAAV[Exp]-TBG>SaCas9, with both constructs packaged into AAV8 viral vectors using medium-scale packaging and ultra-purification services. The CRISPR-Control (CRI-Con) was an EGFP-expressing control AAV8 virus. Mice were administered either CRISPR-Control-AAV8 or CRISPR-Slit2-AAV8 via intravenous injection and maintained for 48 h. Following this period, a single dose of APAP (200 mg/kg) was administered via oral gavage, and mice were sacrificed 24 h after APAP treatment. Blood and liver tissues were collected for further analysis.

### Serum preparation and ALT and AST analysis

After collecting whole blood from mice, the samples were allowed to clot at room temperature for 20 min. The clotted blood was then centrifuged at 9,000 × *g* for 10 min in a refrigerated centrifuge to obtain serum. Serum levels of ALT and AST were measured using commercially available assay kits (ALT: AM102; AST: AM103-K; Asan Pharmaceutical, Hwaseong, Korea) following the manufacturer’s instructions. For the assay, 0.1 mL of the ALT or AST substrate solution was transferred to a test tube and incubated at 37°C for 5 min. Serum samples (0.02 mL) were then added to the substrate solution and incubated at 37°C for 30 min for ALT and 60 min for AST. After incubation, 0.1 mL of the color reagent was added, gently mixed, and allowed to react at room temperature for 20 min. To stop the reaction, sodium hydroxide solution was diluted 10-fold with distilled water, and 1 mL of the diluted solution was added, followed by incubation at room temperature for 10 min. The final reaction mixture (200 μL) was transferred to a 96-well microplate, and absorbance was measured at 505 nm using a microplate reader.

### Histological and IHC staining

Formalin-fixed paraffin-embedded (FFPE) liver tissue sections (4 μm thick) were prepared for histological and IHC analysis. H&E staining was performed to assess histopathological changes, and stained sections were examined using a slide scanner (Aperio CS2; Leica Biosystems, Wetzlar, Germany). For the APAP-induced acute liver injury model, histopathological evaluation of centrilobular necrosis was conducted using a five-grade semiquantitative scoring system based on lesion size and frequency, as follows^53^: grade 0 (no lesion), grade 1 (very mild necrosis), grade 2 (≤30% lobular necrosis), grade 3 (≤60% lobular necrosis), and grade 4 (>60% lobular necrosis). For the TAA-induced subacute liver injury model, histopathological assessment was performed using the Ishak-modified Knodell histological activity index evaluating periportal and periseptal necrosis (PP necrosis), confluent necrosis (C necrosis), and leukocyte infiltration, with PP necrosis graded as grade 0 (no lesion), grade 1 (mild necrosis), grade 2 (mild to moderate necrosis), grade 3 (moderate <50% septa necrosis), and grade 4 (severe >50% septa necrosis); and C necrosis graded as grade 0 (no lesion), grade 1 (focal confluent necrosis), grade 2 (necrosis in some areas), grade 3 (necrosis in most areas), grade 4 (necrosis with occasional portal-central bridging), grade 5 (necrosis with multiple portal-central bridging), and grade 6 (panacinar or multiacinar necrosis).^54^ For IHC staining, FFPE liver tissue sections (4 μm thick) were processed using a Benchmark XT automated IHC stainer (Ventana Medical Systems, Tucson, AZ), and the following primary antibodies were used: SLIT2 (1:200, PA5-31133; Invitrogen, Carlsbad, CA), CD11b (1:600, ab176916; Abcam, Cambridge, UK), ROBO1 (1:100, GTX638936; GeneTex, Irvine, CA), ROBO4 (1:100, BS-5795R; Thermo Fisher Scientific, Waltham, MA), and E-cadherin (1:300, AF748; R&D Systems). IHC results were quantified by calculating the percentage of positive cells in the central vein and pericentral vein region, analyzing four randomly selected high-power fields per sample. IHC staining image acquisition was performed using a Leica Aperio Scanscope CS, and images were analyzed using Aperio ImageScope software (Leica Microsystems). Cell images were obtained using a Leica DMi1 inverted microscope and processed with Leica Application Suite software (Leica Microsystems).

### Dihydroethidium staining

Frozen liver tissue sections from mice treated with APAP or TAA were stained with dihydroethidium (ab236206; Abcam) and DAPI, coverslipped, and incubated for 30 min at 37°C while protected from light. Fluorescence was measured using a confocal microscope (LSM900; Zeiss, Oberkochen, Germany) with an excitation wavelength of 480–520 nm and an emission wavelength of 570–600 nm, and images were analyzed using Zeiss Axio Imager software.

### Cell culture

HepaRG cells (HPRGC10; Thermo Fisher Scientific) were cultured in Williams’ E medium (Gibco, Thermo Fisher Scientific) supplemented with 5× Thaw, Plate & General Purpose medium supplement (HPRG770; Thermo Fisher Scientific) and 5 mL of GlutaMAX supplement (Gibco), or 500 mL of Williams’ E medium with GlutaMAX supplement. HepG2 cells (88065; Korean Cell Line Bank, Seoul, Korea) were maintained in Dulbecco’s modified Eagle’s medium (DMEM; Gibco) supplemented with 10% fetal bovine serum (FBS; Invitrogen) and antibiotics (Invitrogen). AML12 cells (CRL-2254; ATCC, Manassas, VA) were cultured in DMEM/F-12 medium (Gibco) supplemented with 10% FBS (30-2020; ATCC), 10 μg/mL insulin (Sigma-Aldrich), 5.5 μg/mL transferrin (Sigma-Aldrich), 5 ng/mL selenium (Sigma-Aldrich), and 40 ng/mL dexamethasone (Sigma-Aldrich). All cell lines were maintained at 37°C in a humidified incubator with 5% CO_2_.

### TUNEL assay

Apoptotic cell detection was performed using the One-Step TUNEL In Situ Apoptosis kit (E-CK-A320; Elabscience, Houston, TX) according to the manufacturer’s instructions. Cells were seeded onto coverslips, fixed with 1% paraformaldehyde, washed twice with 1× PBS, and treated with 1× permeabilization buffer (00-8333; eBioscience, Thermo Fisher Scientific) for 5 min, followed by additional washing and labeling as per the kit protocol.

### ELISA

Mouse and human serum samples were used for ELISA-based quantification. The following ELISA kits were used: mouse SLIT1 (MBS2020530; MyBioSource, San Diego, CA), mouse SLIT2 (NBP2-82435; Novus Biologicals, Centennial, CO), mouse SLIT3 (MBS2019808; MyBioSource), human SLIT1 (MBS9714247; MyBioSource), human SLIT2 (LS-F4840; LSBio, Seattle, WA), human SLIT3 (MBS2705064; MyBioSource), and mouse IL-6 (KE10007; Proteintech, Rosemont, IL). All assays were performed according to the manufacturer’s instructions.

### MTT assay

Cell viability was evaluated using the MTT Cell Proliferation Assay kit (K299-1000; Biovision, Milpitas, CA) following the manufacturer’s instructions. Cells were seeded in 48-well plates at a density of 3,000 cells per well in 300 μL of culture medium and incubated overnight at 37°C in a humidified atmosphere with 5% CO_2_ to allow for adherence. After MTT treatment as per the manufacturer’s protocol, absorbance was measured at 570 nm using a microplate reader.

### Survival analysis

A total of 14 mice were included in this study, with seven mice per group (control, *n* = 7; rmSLIT2-treated, *n* = 7). Mice were randomly assigned to either the control or rmSLIT2-treated group, receiving i.p. injections of either rmSLIT2 (2 μg/mouse) or 1× PBS twice per week. Survival was monitored over 25 days, with survival status recorded at multiple time points. Kaplan-Meier survival curves were generated to visualize survival probability, and statistical differences between the groups were evaluated using the log-rank test. A *p* value of <0.05 was considered statistically significant. All analyses were performed using GraphPad Prism (version 9.5.1; GraphPad Software, San Diego, CA) or the R survival package.

### CAPE treatment

HepaRG cells were seeded in 6-well plates and cultured until reaching approximately 70%–80% confluence. Cells were treated with CAPE (C8221; Sigma-Aldrich) at concentrations of 20 μM or 40 μM, either alone or in combination with 5 mM APAP. CAPE and APAP were added simultaneously, and cells were incubated for 24 h. Following treatment, total RNA and protein were extracted for real-time PCR and western blot analyses, respectively. Control cells were treated with 0.1% DMSO. All experiments were performed in at least three independent replicates.

### DAS treatment

AML12 cells were seeded in 6-well plates and cultured until reaching approximately 70%–80% confluence. Cells were pretreated with liquid-form DAS (A35801; Sigma-Aldrich) at concentrations of 1.25 μM or 2.5 μM for 2 h, followed by treatment with 3 mM APAP for an additional 24 h. Control cells were treated with 0.1% DMSO. After treatment, whole-cell lysates were harvested for western blotting to evaluate the expression of CYP2E1.

### Fluorescence-activated cell sorting analysis

HepaRG cells were seeded in 6-well plates at a density of 3 × 10^4^ cells per well and treated with rhSLIT2 (200 ng/mL) or APAP (5 mM) for 24 h, either alone or in combination. Intracellular ROS levels were measured using 10 μM 2′,7′-dichlorodihydrofluorescein diacetate (Sigma-Aldrich) in serum-free medium at 37°C for 30 min in darkness. After staining, cells were washed with 1× PBS, harvested, and analyzed using flow cytometry (Aurora 3; Cytek Biosciences, Fremont, CA) with fluorescence detection at excitation 488 nm/emission 525 nm, and data were analyzed using FlowJo software (BD Biosciences).

### ChIP assay

Cells were crosslinked with 1% formaldehyde for 15 min at room temperature on a rocker, quenched with 125 mM glycine for 5 min, pelleted, and resuspended in 500 μL of lysis buffer containing protease inhibitors (K272; Biovision), followed by 10 min of incubation on ice and sonication to shear chromatin. The lysate was then centrifuged at 12,000 × *g* for 10 min at 4°C, and the supernatant was collected, diluted 1:2 with dilution buffer containing protease inhibitors, and incubated overnight at 4°C with 5 μg of NF-κB antibody (51-0500; Invitrogen), followed by the addition of 50 μL streptavidin agarose A beads, rotation at 4°C for 2 h, four washes with prechilled wash buffers, resuspension in 100 μL of chelating resin solution, heating at 95°C for 10 min, centrifugation, collection of the supernatant, rinsing of beads with 100 μL of water, combination of fractions, DNA purification using a DNA purification kit with elution in 50 μL of water, and PCR analysis using 2 μL of purified DNA with the primers presented in Table S3. (These sequences were used to design primers based on information from the JASPAR Transcription Factor Binding Site Database on the UCSC Genome Browser website, indicating that NF-κB binds to each of them).

### NF-κB transcription factor activity assay

HepaRG cells were seeded in 6-well plates at a density of 3 × 10^4^ cells per well, incubated for 24 h, and treated with rhSLIT2 (200 ng/mL) or APAP (5 mM). After treatment, cells were harvested following the Nuclear Extraction Kit (ab113474; Abcam) protocol, and NF-κB transcription factor activity was measured using the NF-κB Transcription Factor Assay kit (ab133112; Abcam) according to the manufacturer’s instructions.

### Western blot analysis

Cells were scraped from plates, and lysates were collected in 1× RIPA buffer (20 mM Tris-HCl [pH 7.5], 150 mM NaCl, 1 mM EDTA, 1 mM EGTA, 1% NP-40, and 1% sodium deoxycholate) containing protease inhibitor cocktail (K272; Biovision) and phosphatase inhibitor cocktail (K282; Biovision). Proteins were separated by SDS-PAGE, transferred onto polyvinylidene fluoride or nitrocellulose membranes, and probed with the following primary antibodies: CYP2E1 (1:3,000, ab28146; Abcam), p-NF-κB (1:1,000, 3033; Cell Signaling Technology), Total NF-κB (1:1,000, 8242; Cell Signaling Technology), IκBα (1:1,000, 4814; Cell Signaling Technology), ROBO1 (1:1,000, PA5-65752; Invitrogen), ROBO4 (1:1,000, ab300046; Abcam), and GAPDH (1:1,000, 60004-1-Ig; Proteintech).

### Real-time PCR

First-strand cDNA was synthesized from 1 μg of total RNA using the RevertAid first-strand cDNA synthesis kit (Thermo Fisher Scientific) with an oligo(dT) primer mix. Real-time PCR was performed using Power SYBR Green PCR Master Mix (Bio-Rad) under the following cycling conditions: initial activation at 95°C for 5 min, followed by 40 cycles of 95°C for 15 s and 60°C for 1 min. The primers used for real-time PCR are presented in Table S4.

### siRNA-mediated gene knockdown

Cells were seeded in 6-well plates at a density of 8 × 10^4^ cells per well and incubated for 24 h. Gene knockdown was performed using siRNA transfection, with 20 μM siRNA stock solutions diluted to a final concentration of 100 nM per siRNA in Opti-MEM (31985070; Gibco) and transfected using Lipofectamine RNAiMAX transfection reagent (13778150; Thermo Fisher Scientific) for 8 h, after which the medium was replaced with complete culture medium. After 48 h, cells were treated with APAP and/or SLIT2 and incubated for an additional 24 h before further analysis. The sequences used for siRNA are presented in Table S5.

### CYP2E1 overexpression

Cells were seeded in 6-well plates at a density of 8 × 10^4^ cells per well and allowed to adhere overnight. The following day, cells were transfected with either an empty control vector or a CYP2E1 overexpression vector, both synthesized by VectorBuilder. Transfections were performed in Opti-MEM (31985-070; Gibco) using Lipofectamine 3000 (L3000-008; Thermo Fisher Scientific) for 8 h, after which the medium was replaced with complete culture medium.

### Lentivirus preparation

Lentiviral particles were generated by co-transfection of the lentiviral expression vector (lentivirus plasmid, shRNA lentiviral vector) with lentiviral packaging plasmids (pGagpol and pVSV-G). HEK-293TN cells were transfected with the lentiviral vectors, and the virus-containing supernatant was harvested after 48 h. Human SLIT2 was cloned and synthesized by VectorBuilder. The coding sequence of SLIT2 was inserted into the pCDH-CMV-MCS-EF1-Puro lentiviral vector (System Biosciences, Mountain View, CA).

### Peptide design using Rfdiffusion

A structural template of the mouse ROBO4 Ig1 domain (UniProt: Q8C310-1; 21–140 amino acids [aa]) in complex with the mouse SLIT2 LRR2 domain (UniProt: Q9R1B9; 291–430 aa) was first generated using AlphaFold3.^55^ AlphaFold3 predictions were performed using default parameters, and the highest-ranked predicted structure was selected and used as the initial template for subsequent peptide design. Peptide design was then carried out using the Rfdiffusion pipeline as previously described,^56^ following the workflow and scripts available at the GitHub repository (https://github.com/nrbennet/dl_binder_design). Structural constraints for Rfdiffusion were defined based on specific interaction hotspots identified on the mouse slit2 LRR2 domain. These hotspots included residues E304, R327-R328, S352, Q375-L376, N399-L400, and Q423-T424, with approximately 2–4 amino acids as spacers between each cluster. Using these constraints and the AlphaFold3-generated template structure, Rfdiffusion generated a total of 5,000 peptide candidates. From these candidates, the top 50 peptides with the highest predicted number of interactions with the Robo4 Ig1 domain were initially selected. Peptides were further filtered by visual inspection of secondary structure predictions and structural modeling, excluding candidates predicted to contain intrinsically disordered regions or structurally unstable motifs. Finally, among structurally favorable peptides, the four peptides predicted to have the highest solubility were selected for further experimental characterization.

### Peptide docking simulations

Docking studies were performed using Discovery Studio 2024 (BIOVIA; Dassault Systèmes, San Diego, CA) to analyze interactions between SP5 (AADGRAAAERRAAALALYGAGALD) and individual mouse ROBO receptor domains ROBO1-Ig1 (UniProt: O89026; 21–120 aa), ROBO1-Ig2 (UniProt: O89026; 122–220 aa), ROBO2-Ig1 (UniProt: Q7TPD3-1; 21–130 aa), ROBO2-Ig2 (UniProt: Q7TPD3-1; 121–220 aa), ROBO3-Ig1 (UniProt: Q9Z2I4-3; 51–160 aa), ROBO3-Ig2 (UniProt: Q9Z2I4-3; 151–250 aa), ROBO4-Ig1 (UniProt: Q8C310-1; 21–140 aa), and ROBO4-Ig2 (UniProt: Q8C310-1; 131–230 aa). Structural models were predicted using AlphaFold3.^55^ All protein and peptide structures underwent preparation by applying pH 7.4 protonation and the CHARMm force field as a preprocessing step before docking. Initial docking was executed using the ZDOCK protocol with default parameters. The poses generated from initial docking were clustered based on similarity in their binding pocket locations. Clusters relevant to the known Robo-Slit binding pocket structures were manually selected, and all poses within these selected clusters underwent refinement using the RDOCK protocol with default parameters. The final top-ranked pose was identified based on the highest RDOCK score after energy minimization.

### RNA-seq analysis

Total RNA was extracted from liver tissue samples from two independent biological replicates using the Macherey-Nagel RNA kit (Macherey-Nagel, Düren, Germany), and RNA integrity was assessed using a Bioanalyzer RNA Chip (Agilent Technologies, Santa Clara, CA). RNA-seq was performed using the NextSeq 500 platform (Illumina, San Diego, CA), and sequencing libraries were prepared by LAS (Gimpo, Korea). To process sequencing data, Skewer (v.0.2.2) was used for adapter trimming and removal of low-quality bases from raw reads, and high-quality reads were mapped to the reference genome using STAR (v.2.5). Gene expression was quantified in fragments per kilobase of transcript per million mapped reads (FPKM) using gene models based on the mm39 reference genome annotation (UCSC genome, GTF format). Heatmaps were generated using FPKM values to visualize gene-expression patterns. For variance stabilizing transformation and gene set enrichment analysis (GSEA), raw sequencing data were trimmed and mapped using AltAnalyze (v.2.1.4.3). Raw count data were analyzed using the DESeq2 package (v.1.38.3) in R (v.4.0.3), with differential expression analysis performed using the DESeq function and sample distance calculated from VST-transformed data, visualized with the pheatmap function and ggplot function. GSEA was conducted using the fgsea package (v.1.24.0) in R, with 1,000 permutations and *p* values computed using GSEA statistics, where the weighted Kolmogorov-Smirnov statistic was applied to a running sum of a ranked gene list.

### Analysis of public RNA-seq analyses

Publicly available bulk RNA-seq datasets (GEO: GSE218879, GSE243679, and GSE195753) were used to analyze gene-expression changes in mouse liver injury models. These datasets contain FPKM-normalized RNA-seq data obtained from mouse liver tissues following APAP treatment for 6 h. Boxplots and heatmaps were generated using FPKM values to visualize gene-expression patterns and variations across different conditions.

### IPA

IPA (QIAGEN, Redwood City, CA) was used to identify transcriptional regulators and pathway alterations associated with APAP-induced liver injury and Slit2-mediated modulation. Differentially expressed genes (DEGs) from APAP vs. control and rmSlit2 + APAP vs. APAP comparisons were uploaded to IPA for core analysis. The analysis parameters were set with a log_2_ fold-change cutoff of ≤ −0.05 (downregulated) and ≥0.05 (upregulated) with *p* ≤ 0.05. For transcriptional regulator analysis, APAP vs. control DEGs were ranked based on activation *Z* score (≥2.5), indicating significant transcriptional activation, and the identified transcription factors were compared with SLIT2 + APAP vs. APAP DEGs to assess regulatory differences. Canonical pathway enrichment, upstream regulator analysis, and network prediction were performed using IPA’s curated knowledge base. The activation or inhibition of predicted pathways was determined based on *Z-*score calculations, where Z ≥ 2 indicates activation and Z ≤ −2 indicates inhibition.

### Statistical analysis

Numerical data are expressed as median with interquartile range (IQR) for non-normally distributed datasets and as mean ± standard deviation (SD) for normally distributed datasets. Comparisons between control and experimental groups were performed using either the Student’s *t* test for normally distributed data or the one-tailed Mann-Whitney U test for non-normally distributed data, with the one-tailed approach applied when a directional change was specifically hypothesized. All statistical analyses were performed using GraphPad Prism v.9.5.1 (GraphPad Software), and exact *p* values are reported in the figure panels or legends. A *p* value of <0.05 was considered statistically significant.

## Data and code availability

The data presented in this study are publicly available in NCBI’s Gene Expression Omnibus and Metabolomics Workbench under the accession codes GEO: GSE18879 (https://www.ncbi.nlm.nih.gov/geo/query/acc.cgi?acc=GSE18879), GSE243679 (https://www.ncbi.nlm.nih.gov/geo/query/acc.cgi?acc=GSE243679), GSE195753 (https://www.ncbi.nlm.nih.gov/geo/query/acc.cgi?acc=GSE195753), and GSE291946 (https://www.ncbi.nlm.nih.gov/geo/query/acc.cgi?acc=GSE291946).

## Acknowledgments

These experiments were supported by the 10.13039/501100003725National Research Foundation of Korea (NRF-2019R1A2C2086127, NRF-2020R1A6A1A03043539, and NRF-2020M3A9D8037604 to T.J.P.; NRF-2021R1I1A1A01058774 to Y.H.K.; NRF-2022R1I1A1A01072350 to J.H.C.), 10.13039/501100025314Ajou University School of Medicine (M-2024-C0460-00103), and the 10.13039/501100003710Korea Health Industry Development Institute (KHIDI) funded by the Ministry of Health & Welfare, Republic of Korea (RS-2024-00440193 and HR22C1734 to Y.W.C.).

## Author contributions

Y.H.K. and Y.W.C. contributed to study design, data collection, analysis, interpretation, and manuscript drafting; Y.W.C. and J.H.C. contributed to data interpretation and methodology; S.H.P. performed histological analyses; Y.-S.L., J.J., and E.K. were responsible for peptide synthesis and related data analysis; Y.H.K., Y.W.C., and T.J.P. jointly led the study, including conceptualization, funding acquisition, supervision, and data interpretation; and Y.-K.L. and S.S.P. carried out the revision cell-culture work and IHC imaging; H.Y.K. provided research advice and supervised the study.

## Declaration of interests

The authors declare no competing interests.

## Supplemental information

Document S1. Figures S1–S11 and Tables S1–S5

Table S6. Source data supporting all figures and supplemental figures in the manuscript

Document S2. Article plus supplemental information
