# Supplementary material for: SLIT2 as a key regulator and therapeutic target in liver injury
Source: Mol Ther. 2026 Jan 10;34(4):2446–67. doi: 10.1016/j.ymthe.2026.01.010 (PMC13069340; doi:10.1016/j.ymthe.2026.01.010)
Supplement: Supplementary file 1 [file mmc1.pdf]

YMTHE, Volume *34*

Supplemental Information

SLIT2 as a key regulator

and therapeutic target in liver injury

Yong Won Choi, Jae Ho Choi, Young-Sam Lee, Jinju Jeong, Eunho Kang, So Hyun Park, Young-Kyoung Lee, Soon Sang Park, Hee Young Kang, Young Hwa Kim, and Tae Jun Park

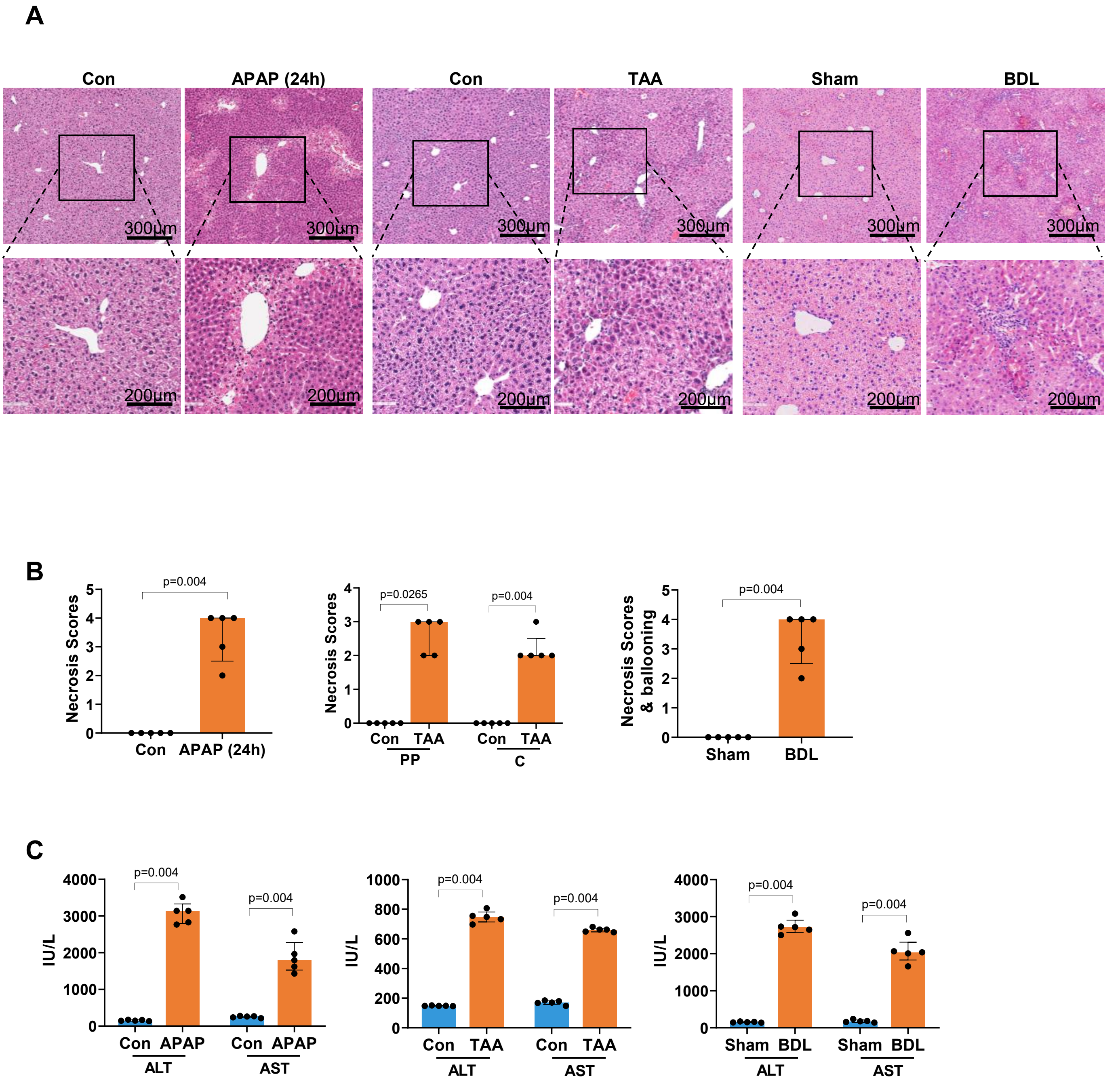

Figure S1. Validation of Liver Injury Models Induced by APAP, TAA, and BDL. (A) Representative H&E-stained liver sections from control (Con) and injury groups (APAP, TAA, BDL). The Sham group serves as the control for the BDL model. Dashed lines indicate necrotic regions. Scale bars, 200–300 μm as indicated. (B) Quantification of necrotic areas on H&E (n = 5 per group). (C) Serum ALT and AST levels for each model (n = 5 per group). Statistical significance was determined using the Mann-Whitney U test. Data is presented as median ± IQR.

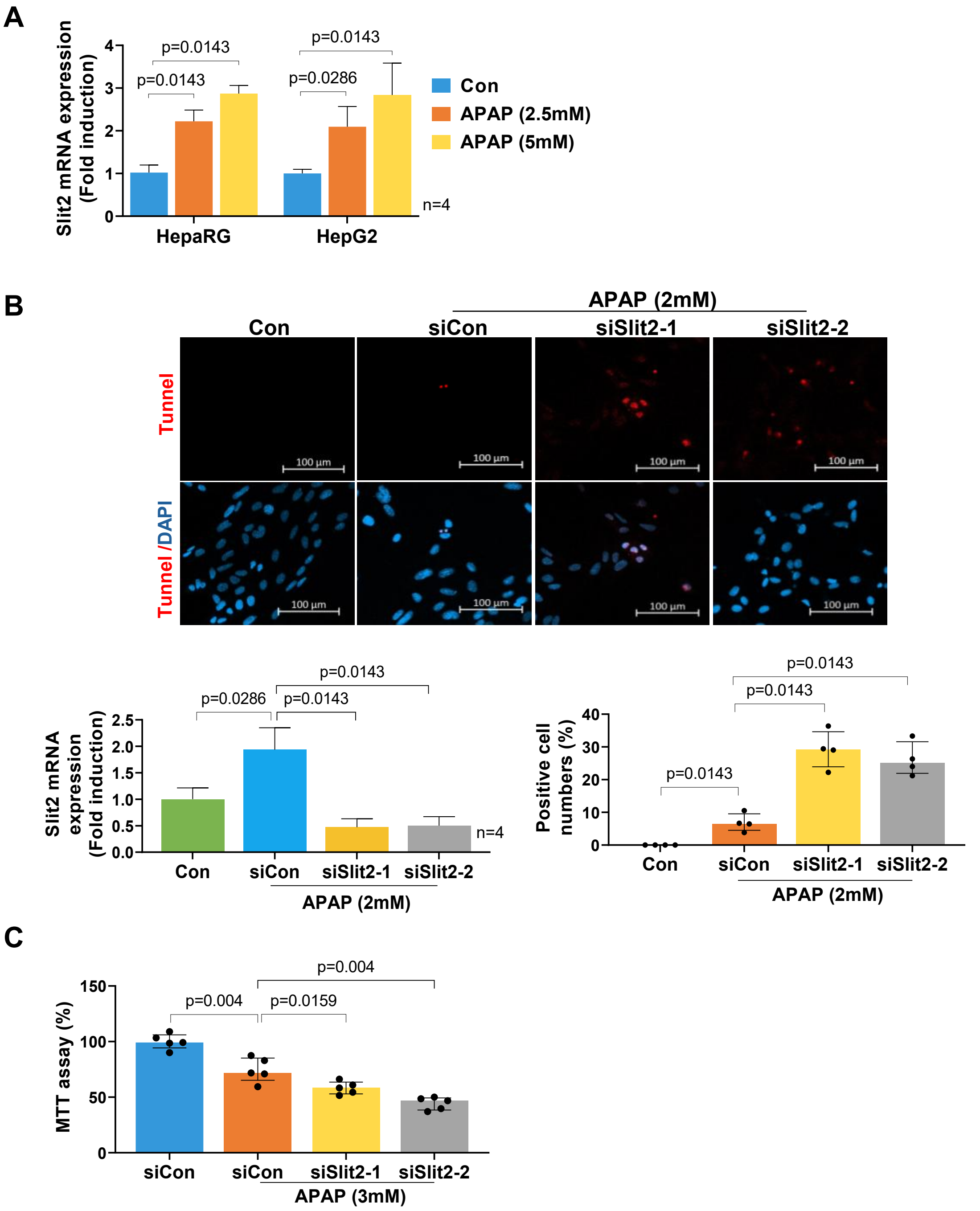

Figure S2. SLIT2 protects hepatic cells from APAP-induced cytotoxicity. (A) *Slit2* mRNA expression in HepaRG and HepG2 cells following APAP (2.5 mM, 5 mM) treatment for 24 h, quantified by real-time PCR. (B) TUNEL assay. HepaRG transfected with siControl (siCon), siSlit2-1, or siSlit2-2 were treated with APAP (2 mM) for 48 h. The upper panel presents representative TUNEL-stained images. The lower left panel quantifies *Slit2* mRNA expression by real-time PCR. The lower right panel quantifies TUNEL-positive cells (four randomly selected fields per sample, ×20 magnification). (C) MTT assay. HepaRG cells were transfected with siCon, siSlit2-1 or siSlit2-2 and then treated with APAP (3 mM) for 48 h. Statistical significance was determined using the Mann-Whitney U test. Data is presented as median ± IQR.

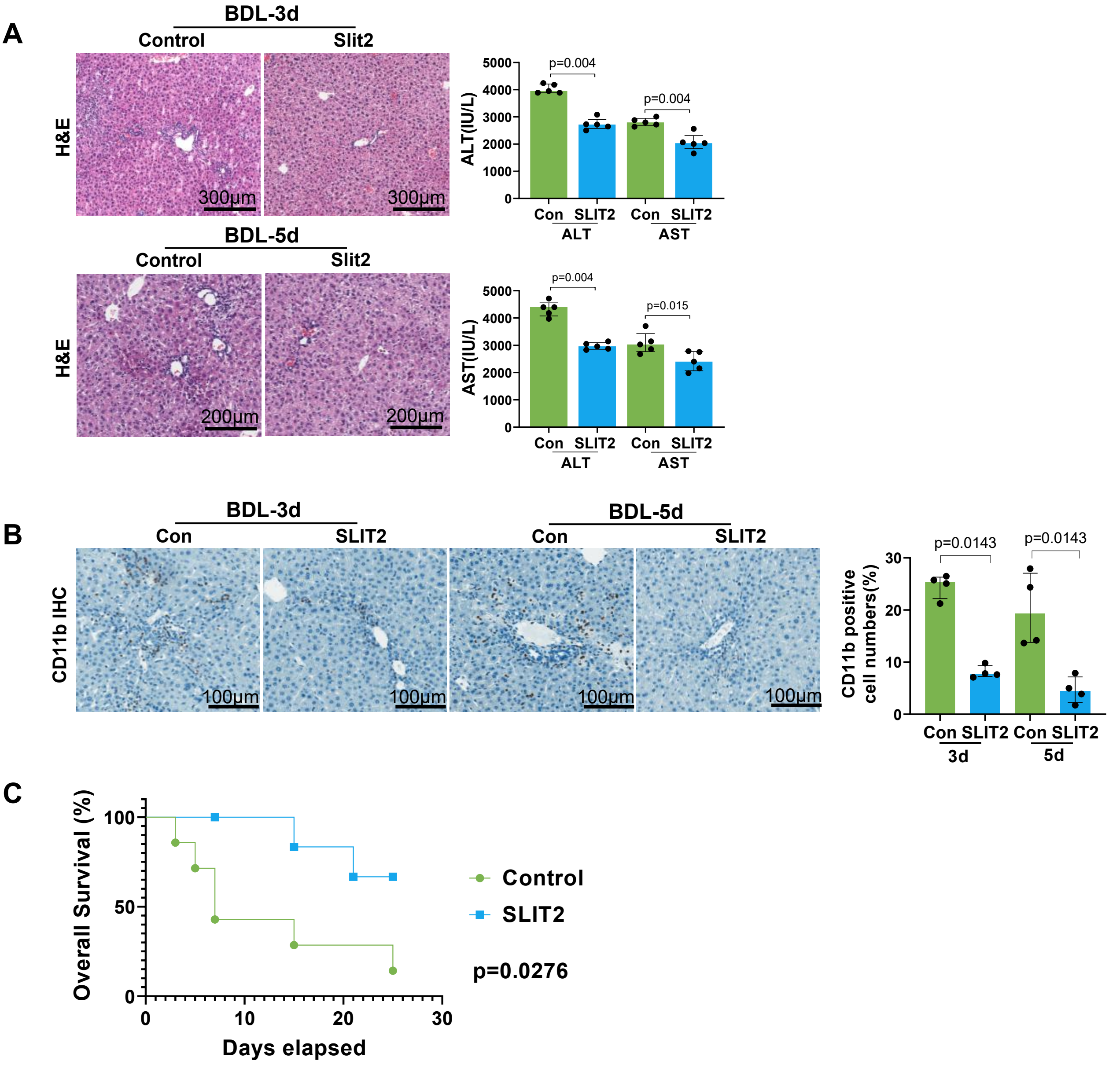

Figure S3. SLIT2 protects against BDL-induced liver injury and improves survival. (A) SLIT2 treatment in BDL-induced liver injury. Mice underwent BDL surgery and were treated rmSLIT2 (2 µg/mouse) or PBS immediately post-surgery. Mice were sacrificed at 3 and 5 days post-BDL. Representative H&E-stained liver sections and serum ALT/AST levels are shown (n = 5 per group). (B) CD11b-positive immune cell infiltration in liver tissues following BDL. Quantification was performed from four randomly selected image fields per condition. (C) Survival analysis following BDL or BDL/rmSLIT2 treatment. Mice were treated twice weekly with rmSLIT2 (2 µg/mouse) or PBS following BDL, and survival rate was measured over 25 days. Data is presented as median ± IQR. Statistical significance was determined using the Mann-Whitney U test.

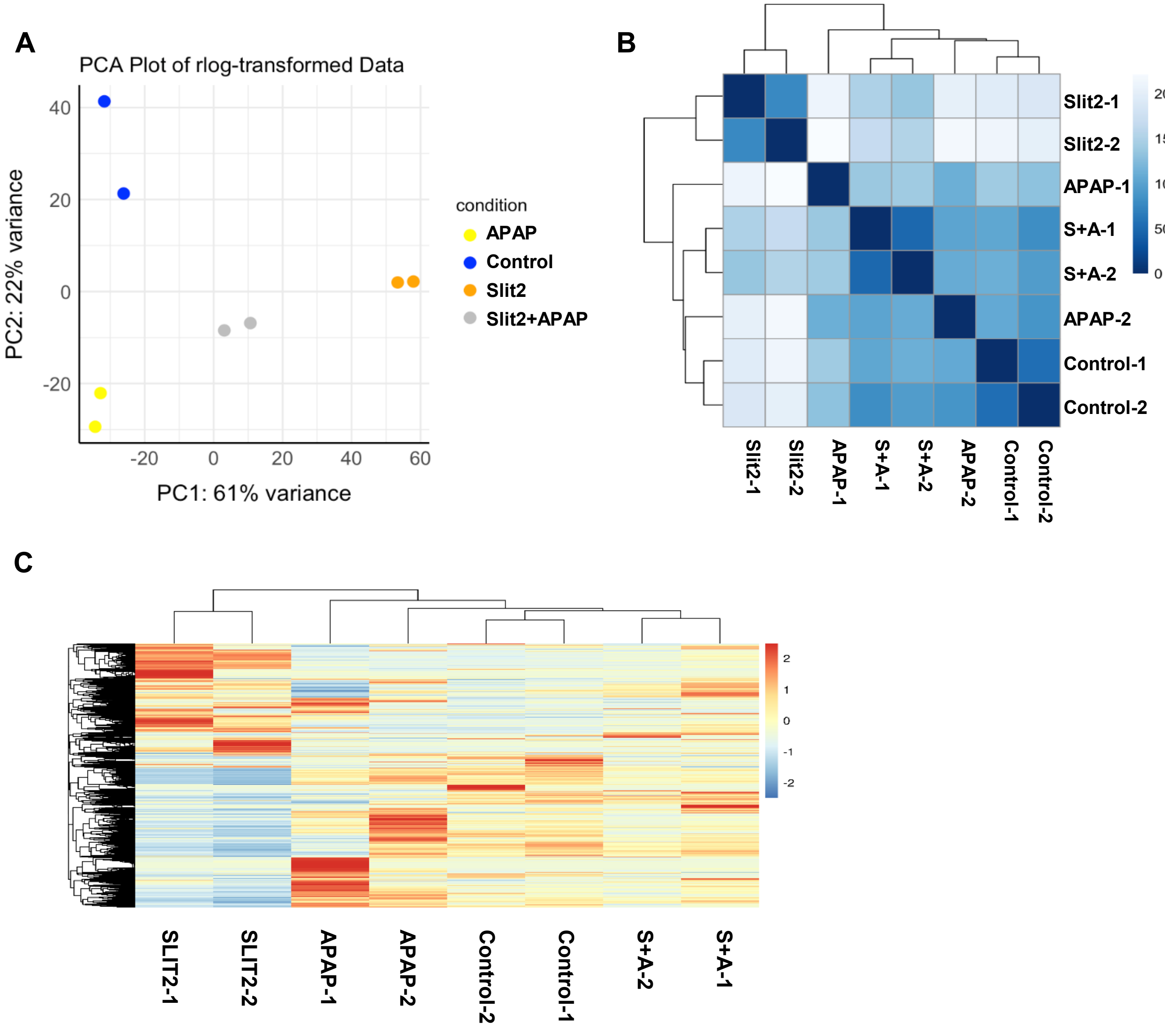

Figure S4. Transcriptomic Profiling Reveals Distinct Expression Patterns Across Experimental Groups. (A) Principal Component Analysis (PCA) was performed on RNA-Seq transcriptomic data to assess the global gene expression variability across Control, APAP, rmSLIT2, and rmSLIT2+APAP groups. The PCA plot visualizes sample distribution in a two-dimensional space, with principal component 1 (PC1) and principal component 2 (PC2) representing the highest variance in gene expression profiles. Each point represents an individual sample, and clustering patterns reflect transcriptional similarities and differences among treatment groups. (B) A heatmap with hierarchical clustering was generated to visualize sample-to-sample correlations based on RNA-Seq transcriptomic profiles across different conditions, including Control, APAP, rmSLIT2, and rmSLIT2+APAP. The color gradient represents pairwise similarity values, with darker blue indicating higher correlation and lighter blue indicating lower correlation between samples. (C) A clustering heatmap representing gene expression profiles across different experimental conditions. The heatmap shows normalized expression values (z-scores) with red indicating upregulated genes, blue indicating downregulated genes, and yellow representing moderate expression levels. The x-axis represents different experimental groups: SLIT2-1, SLIT2-2, APAP-1, APAP-2, Control-1, Control-2, SLIT2+APAP-1 (S+A-1), and SLIT2+APAP-2 (S+A-2), while the y-axis represents individual genes. Dendrograms at the top illustrate hierarchical clustering of samples based on gene expression similarities.

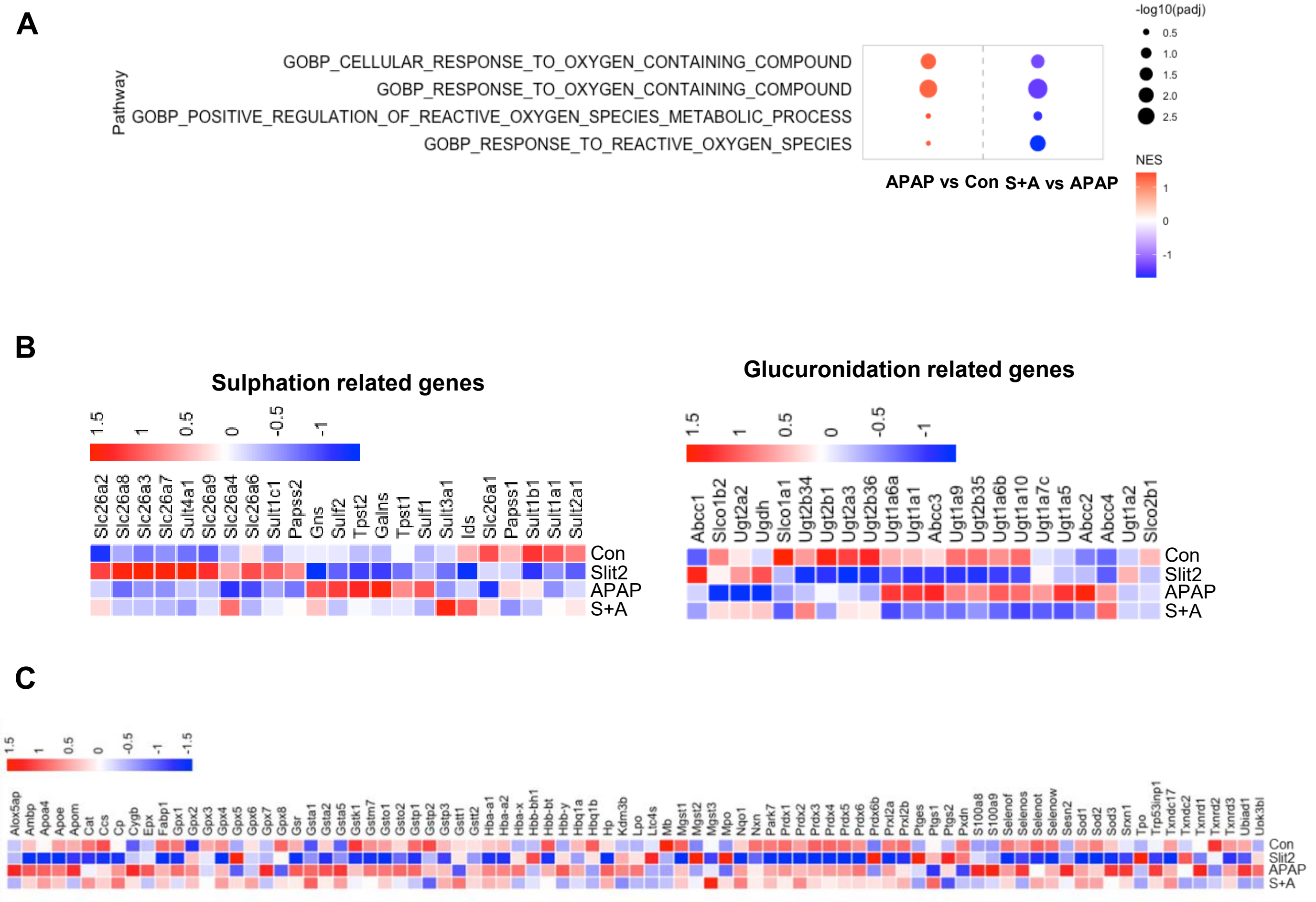

Figure S5. Transcriptomic Signatures of Oxidative Stress, Transport, and Antioxidant Pathways in Response to SLIT2 and APAP Treatment (A) A dot plot was generated to visualize the Gene Ontology (GO) enrichment analysis of oxidative stress-related biological processes. The y-axis lists significantly enriched GO pathways, while the x-axis represents enrichment ranking. The dot size corresponds to -$\log_{10}$(adjusted p-value), reflecting statistical significance, and the color gradient represents the normalized enrichment score (NES), with darker blue indicating stronger negative enrichment. (B) Heatmaps display the expression profiles of genes related to sulphation (left) and glucuronidation (right) in Control, rmSLIT2-treated, APAP-treated, and rmSLIT2+APAP-treated (S+A) liver samples. Gene expression levels are z-score normalized, with a color scale indicating upregulation (red) and downregulation (blue). The left heatmap includes solute carrier (Slc) and sulfotransferase (Sult) family genes, while the right heatmap shows UDP-glucuronosyltransferase (Ugt) and ATP-binding cassette (Abc) transporter genes. (C) A heatmap was generated to visualize the expression profiles of antioxidant-related genes associated with Gene Ontology Molecular Function (GOMF) Antioxidant Activity in Control, rmSLIT2-treated, APAP-treated, and rmSLIT2+APAP-treated (S+A) liver samples. Gene expression levels are z-score normalized, with a red-to-blue color gradient representing upregulation (red) and downregulation (blue).

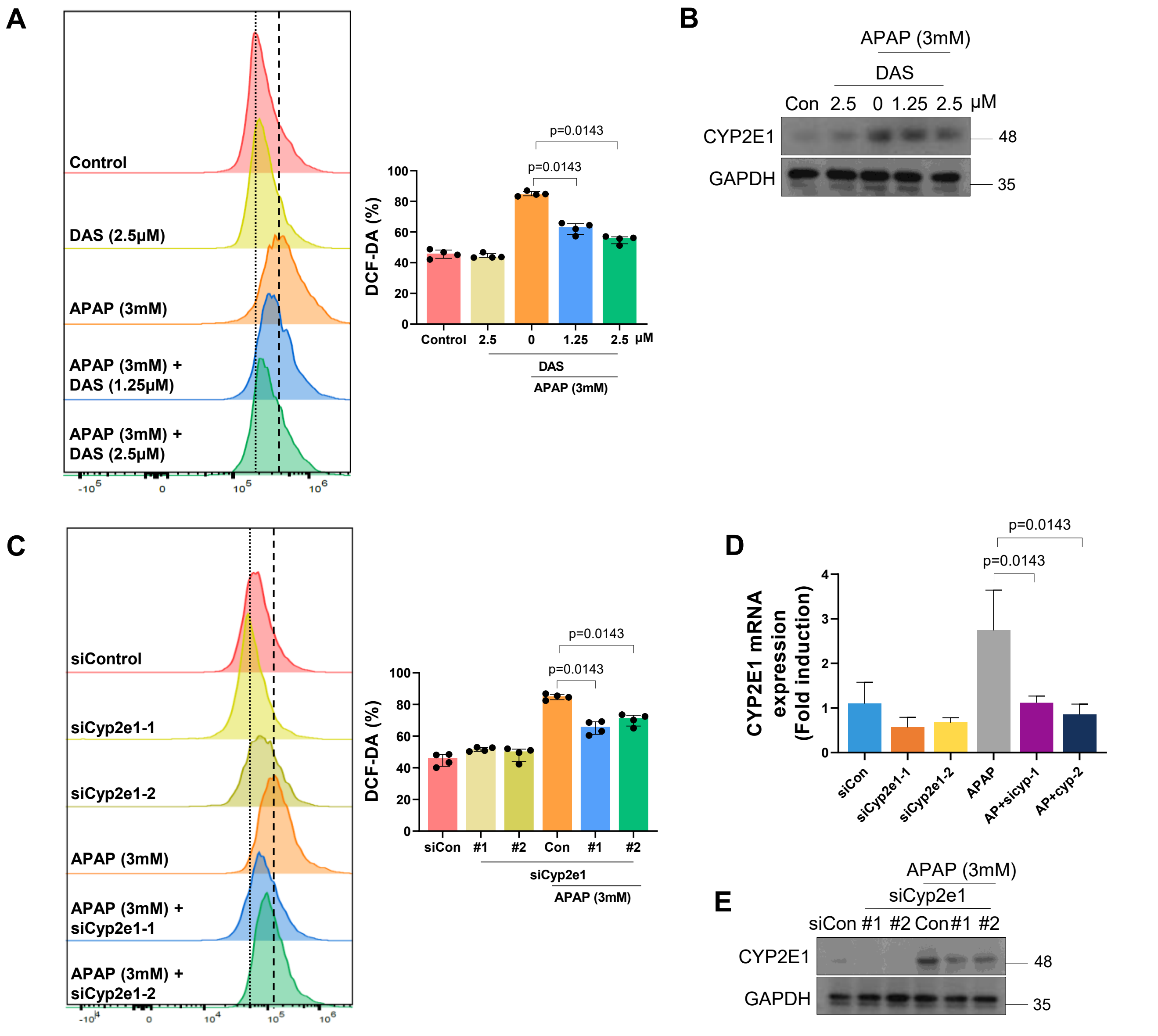

Figure S6. Suppression of CYP2E1 Attenuates ROS Generation in AML12 Cells upon APAP Exposure. (A–B) Diallyl sulfide (DAS) attenuates APAP-induced ROS and CYP2E1 expression. AML12 cells were treated with 3 mM APAP with or without DAS (1.25 or 2.5 μM) for 24 h. (A) ROS levels were measured by DCF-DA flow cytometry. Overlaid histograms show representative fluorescence intensity distributions; bar graph summarizes quantification (n = 4). (B) Western blot analysis of CYP2E1 protein levels with GAPDH as a loading control. (C–E) siRNA-mediated knockdown of CYP2E1 reduces APAP-induced ROS. Cells were transfected with siControl or two independent siRNAs (siCyp2e1-1, siCyp2e1-2), followed by APAP (3 mM, 24 h). (C) Representative ROS histograms and quantification (n = 4). (D) qRT-PCR validation of CYP2E1 mRNA knockdown normalized to GAPDH. (E) Western blot confirmation of reduced CYP2E1 protein expression. Data are presented as median ± IQR; $p < 0.05$ by Mann–Whitney U test.

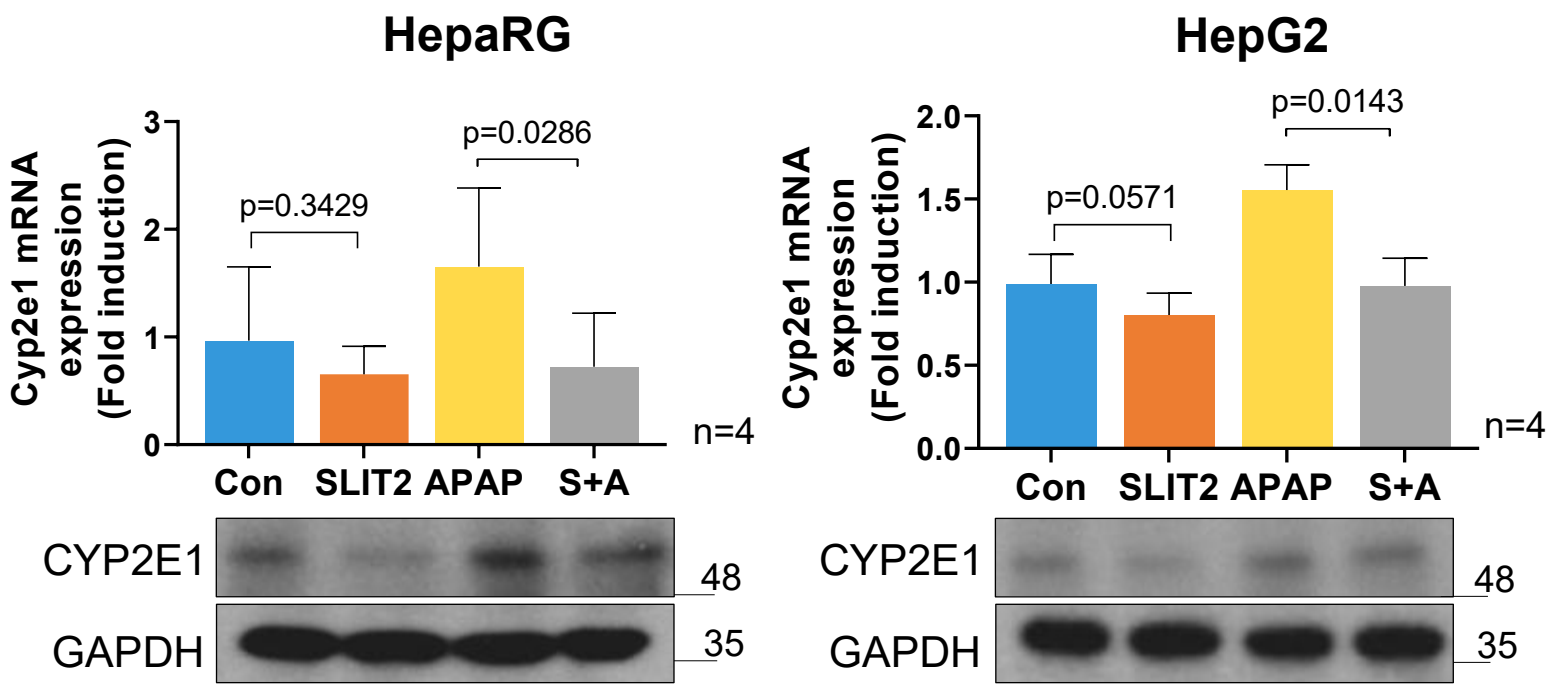

Figure S7. SLIT2 suppresses APAP-induced CYP2E1 expression in human hepatic cells. *CYP2E1* expression in HepG2 and HepaRG cells treated for 24 h with control (CON), APAP (5 mM), rhSLIT2 (200 ng/mL), or APAP + rhSLIT2 (S+A). Expression levels were assessed at both mRNA and protein levels using real-time PCR and western blotting, respectively. Data is presented as median ± IQR. Statistical analysis was performed using the Mann–Whitney U test.

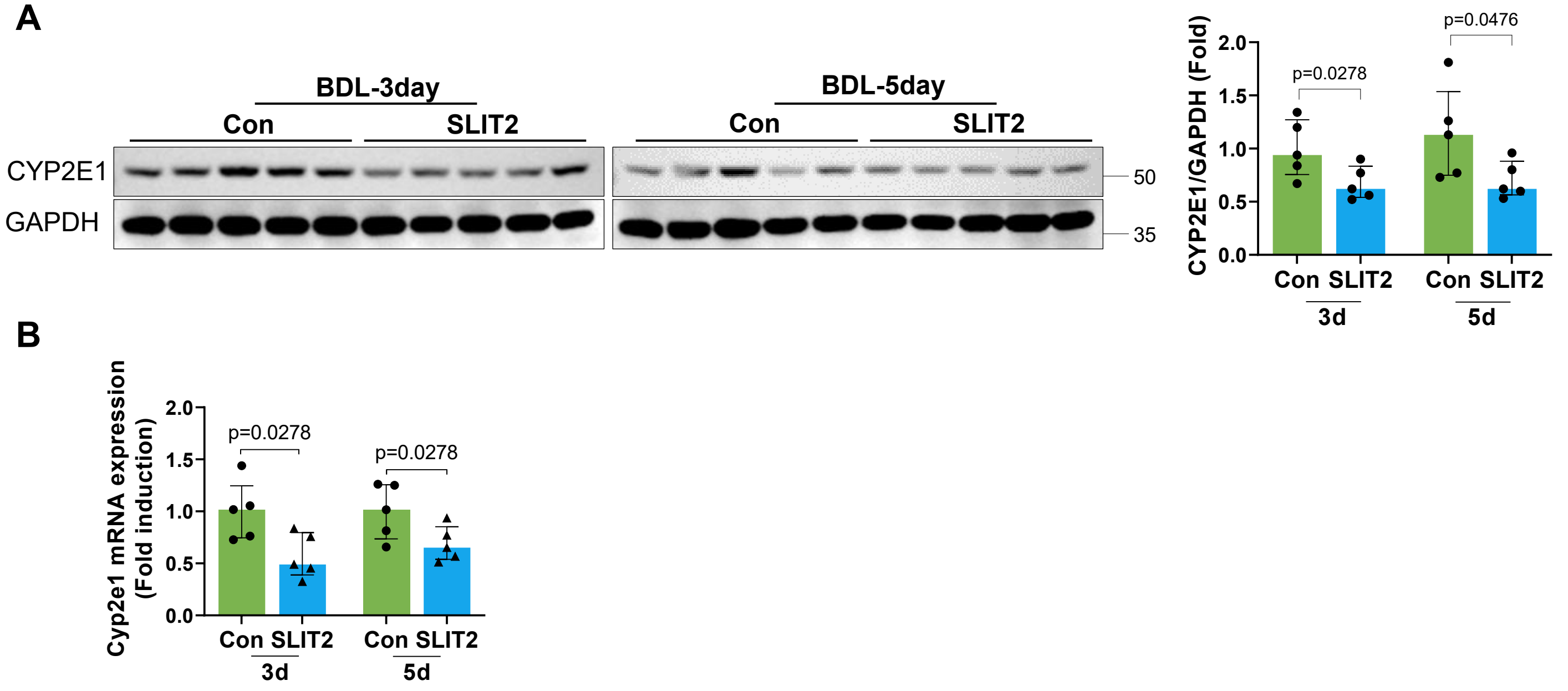

Figure S8. SLIT2 reduces CYP2E1 expression in BDL-induced liver injury. (A) Western blot analysis of CYP2E1 protein expression in BDL mice liver. Protein expression was evaluated at 3 days (BDL 3d) and 5 days (BDL 5d) after BDL. The right panel presents densitometric quantification of CYP2E1 protein expression (n = 5 per group). (B) Real-time PCR analysis of *Cyp2e1* mRNA expression in BDL mice liver tissues at 3 and 5 days post-BDL. Data are presented as fold change relative to control (n = 5 per group). Data is presented as median ± IQR. Statistical analysis was performed using the Mann–Whitney U test.

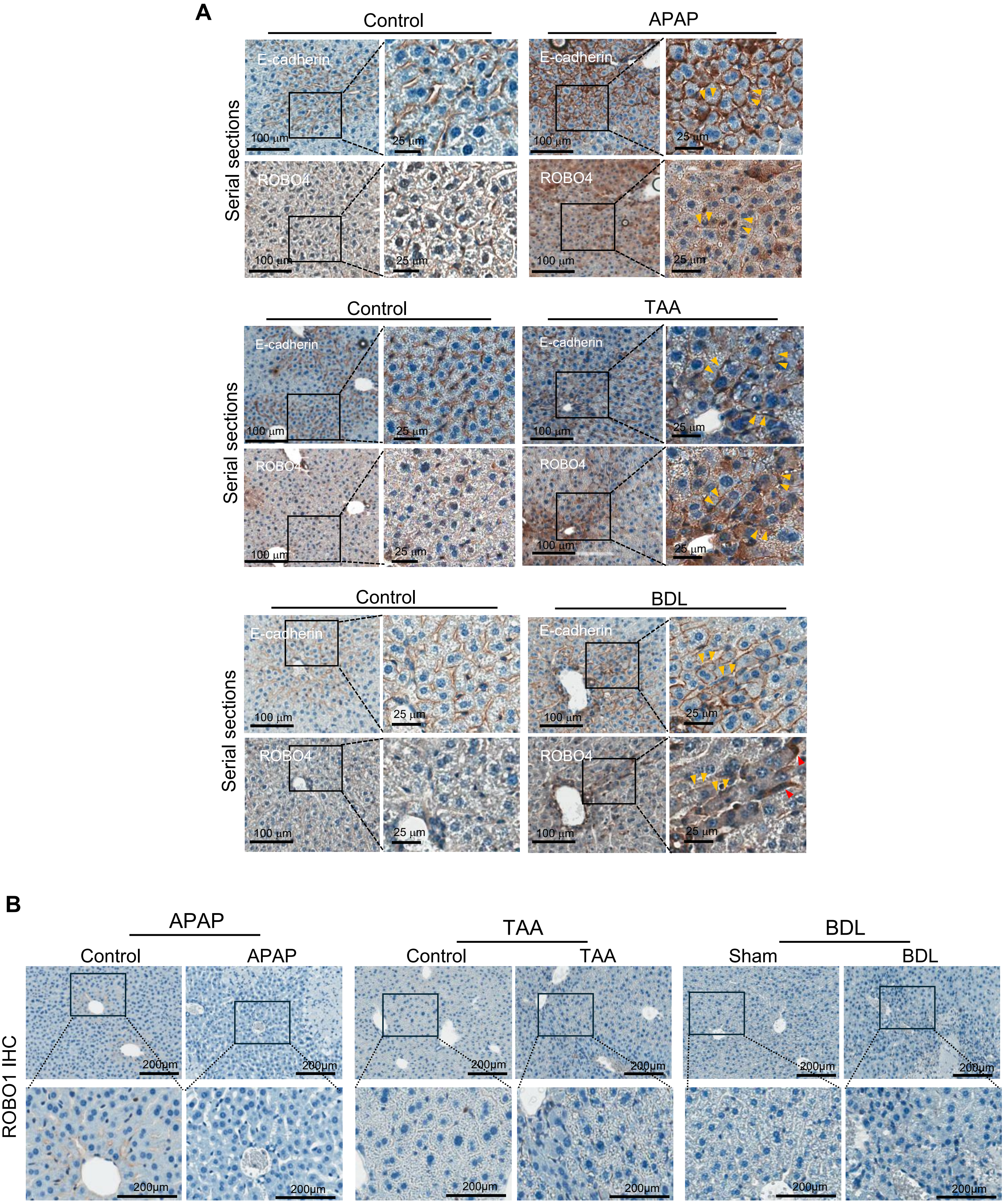

Figure S9. Hepatic ROBO4 upregulation in multiple liver injury models and comparison with ROBO1 expression. Representative immunohistochemical staining for ROBO4 (A) and ROBO1 (B) in liver tissues from Control, or APAP-, TAA-, and BDL-treated mice. Insets show magnified views of boxed regions to visualize hepatocyte morphology. (A) Yellow arrowheads indicate E-cadherin or ROBO4 staining localized to the plasma membrane, and red arrowheads indicate ROBO4 staining in non-parenchymal cells observed in the BDL model. All liver injury models used in this figure were validated as shown in Supplementary Figure S1.

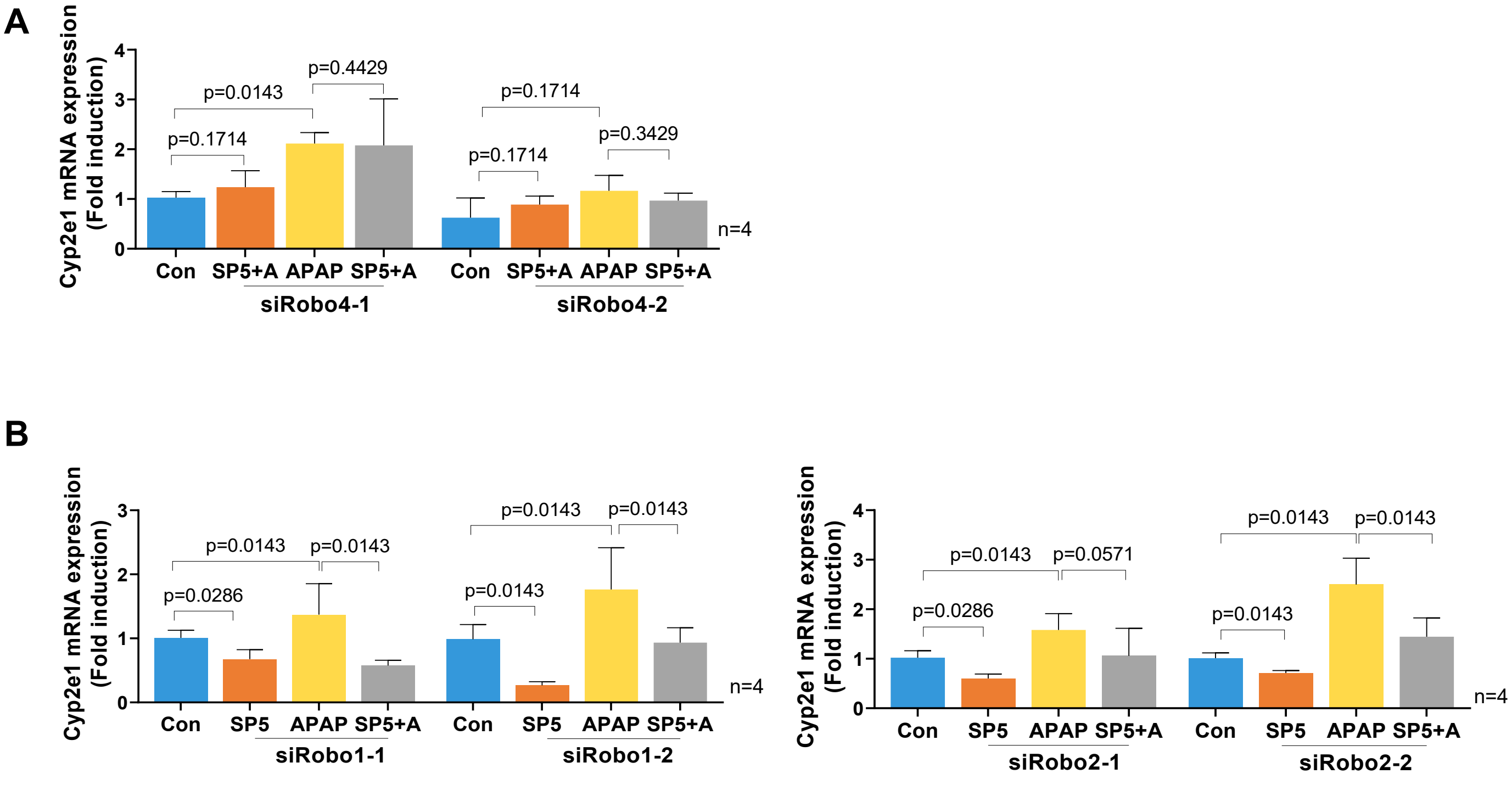

Figure S10. **ROBO4 is specifically required for SP5-mediated suppression of Cyp2e1 expression.** (A) AML12 cells were transfected with siRobo4, followed by treatment with SP5 and/or APAP. Cyp2e1 mRNA expression was analyzed using real-time PCR. (B) Knockdown of Robo1 or Robo2 does not affect SP5-mediated Cyp2e1 suppression. AML12 cells were transfected with siRobo1 or siRobo2, followed by treatment with SP5 and/or APAP. Cyp2e1 mRNA expression was analyzed using real-time PCR. Data is presented as median ± IQR. Statistical analysis was performed using the Mann–Whitney U test.

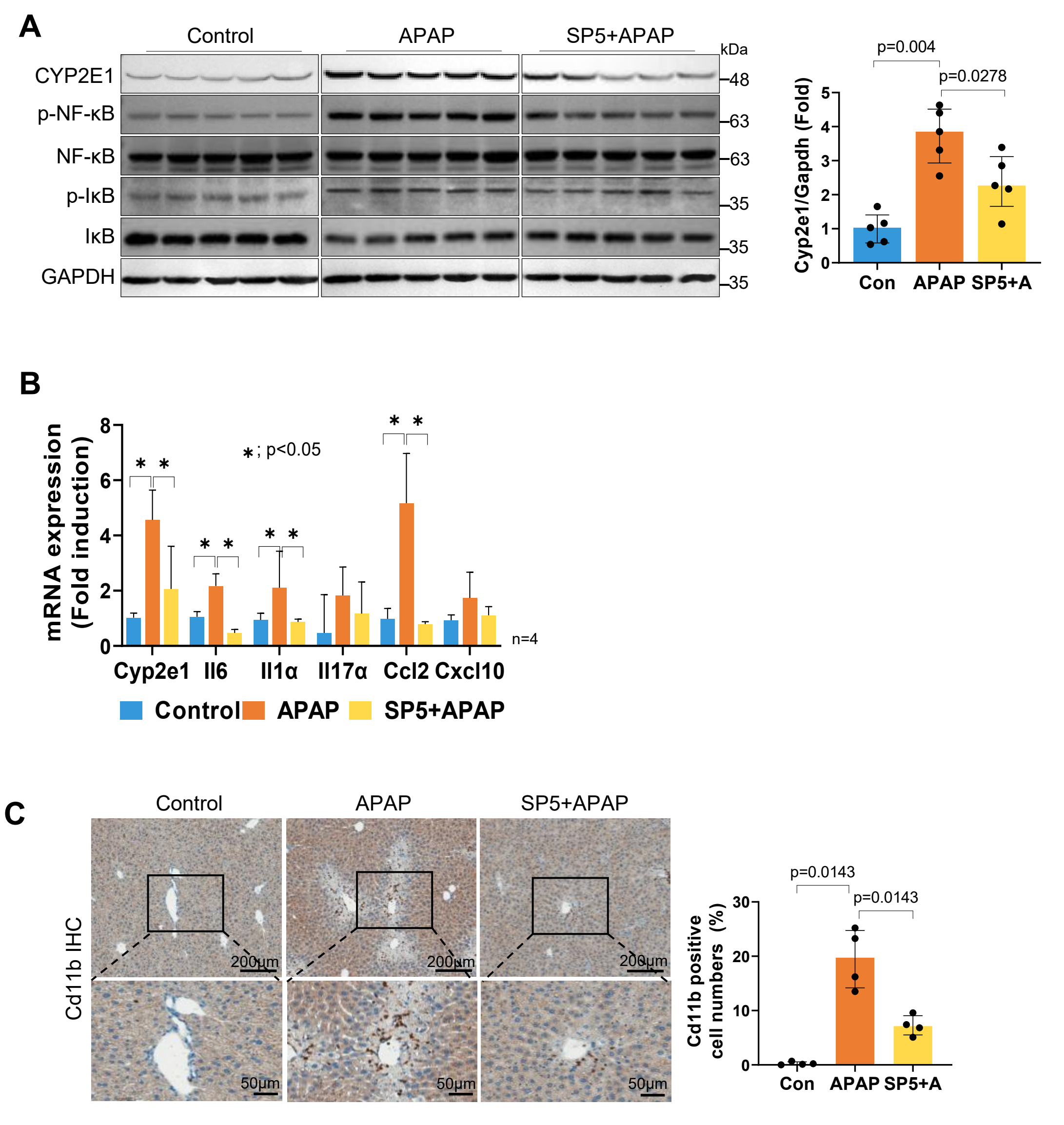

Figure S11. **SP5 post-treatment blocks NF-κB activation and reduces inflammation in APAP-induced liver injury.** (A) Western blot analysis of p-NF-κB, p-IκB and CYP2E1 protein expression in liver tissues. Right panel: densitometric quantification normalized to GAPDH. (B) Real-time PCR analysis of inflammatory gene expression in liver tissues following SP5 treatment, performed with four independent replicates. (C) CD11b immunohistochemistry in liver tissues. Left: representative images. Right: quantification of $CD11b^+$ cell infiltration based on four randomly selected fields per condition. Data are presented as median ± IQR and statistical significance was determined using the Mann–Whitney U test.

Table S1. Docking scores obtained from ZDOCK and RDOCK analysis of the interaction between peptide #5 and Ig domains of Robo proteins.

mRobo	Ig Domain	ZDOCK (2000pose) and RDOCK(clustering and refine)							
		ZDock score	ZRank score	E_vdw1	E_elec1	E_vdw2	E_elec2	E_sol	E_Rdock
mRobo1	Ig1	9.68	-63.222	-53.9684	-1.45297	-58.4013	-1.75478	-9.3	**-10.8793**
mRobo1	Ig2	10.14	-16.363	-45.2563	1.1895	-51.3793	-1.03773	1.5	**0.56604**
mRobo2	Ig1	9.64	-54.286	-30.4233	-2.68467	-56.257	-1.12836	-10.7	**-11.7155**
mRobo2	Ig2	9.28	-38.801	-42.5384	0.273198	-41.2509	-0.32236	0.6	**0.309878**
mRobo3	Ig1	10.38	-27.904	-68.4868	-1.97875	-69.0791	-5.87548	-5.3	**-10.5879**
mRobo3	Ig2	9.34	-63.256	-44.5674	0.252016	-55.0334	0.707474	-12.3	**-11.6633**
mRobo4	Ig1	9.58	-53.042	-41.9192	0.540967	-45.9604	-6.53616	-7.6	**-13.4825**
mRobo4	Ig2	9.02	-47.91	-51.7509	-1.92096	-51.6892	-0.44744	-7	**-7.40269**

A molecular docking analysis was performed to evaluate the binding interaction between SP5 and the Ig1 domain of mROBO4 using ZDOCK (2000 poses) and RDock refinement. The table presents docking scores, energy components, and binding affinity parameters, including van der Waals energy (E_vdw), electrostatic energy (E_elec), solvation energy (E_sol), and overall refined docking energy (E_Rdock). The results indicate a favorable binding interaction between SP5 and the mROBO4 Ig1 domain, with a ZRank score of -53.042 and an E_Rdock value of -13.4825, suggesting a stable binding conformation that may underlie SP5-mediated ROBO4 activation.

Zdock; The Dock Proteins (ZDOCK) protocol provides rigid body docking of two protein structures using the ZDOCK algorithm[Chen and Weng, 2002] as well as clustering the poses according to the ligand position. Optionally, the docked poses can be filtered for complexes that include a user specified set of residues at the binding interface. In addition, the protocol provides the option of reranking the docked poses with the ZRANK scoring function. ZDock Score; ZDock Score is the shape complementarity score calculated by the ZDOCK program. If the Use Electrostatic and Desolvation Energy advanced protocol parameter is set to True, the ZDOCK score also includes electrostatics and desolvation energy terms. Higher scores are better. ZRank Score; ZRank Score is the energy of the docked posed calculated by the ZRANK rescoring method. This column is only present if the ZRank protocol parameter is set to True. Lower scores are better. Rdock; Refine Docked Proteins (RDOCK) provides optimization and scoring of a set of docked protein poses generated by the Dock Proteins (ZDOCK) protocol - a rigid docking method, using a CHARMm minimization. E_Elec1; Electrostatic energy after first minimization with ionic residues in neutral state. E_Elec2; Electrostatic energy after second minimization with ionic residues in charged state. E_Sol; Desolvation energy based on the Atomic Contact Energy (ACE). E_vdw1; van der Waals energy after first minimization with ionic residues in neutral state. E_vdw2; van der Waals energy after second minimization with ionic residues in charged state. clash Flag specifying if there are van der Waals clashes for the pose (defined as E_vdw2 > 10 kcal/mol). E_Rdock; Final RDOCK energy: E_RDock=E_sol+beta*E_elec2.

Table S2. Human serum information in this study.

Case number	Gender	Age	Clinical diagnosis	Cause of hepatitis	Creatinine	eGFR	T.cholesterol	GOT	GPT	r-GTP	WBC	Hb	PLT	SLIT1 (pg/mL)	SLIT2 (ng/mL)	SLIT3 (ng/mL)
Ajou-p1	M	26	toxic hepatitis	Medication	0.8	≥60.00	132	170	675	137	4.4	14.9	300	0	1.598	0.075
Ajou-p2	F	62	toxic hepatitis	Medication	0.77	≥60.00	166	28	18	44	5.7	13.7	249	0	0.506	0.362
Ajou-p3	M	52	toxic hepatitis	Medication	1.06	≥60.00	127	35	45	31	7.1	15.7	185	0	0.172	0
Ajou-p4	M	35	toxic hepatitis	Medication	0.9	≥60.00	99	58	44	93	5.1	15.0	280	0	0.272	0
Ajou-p5	M	45	toxic hepatitis	Medication	0.92	≥60.00	282	28	12	377	6.0	15.9	235	0	0.17	0
Ajou-p6	F	37	toxic hepatitis	herbal medicine	0.57	≥60.00	101	3251	1836	228	1.3	13.9	205	26.049	0.174	0
Ajou-p7	F	49	toxic hepatitis	Medication	0.71	≥60.00	232	169	293	97	8.0	13.8	287	0	0.156	0
Ajou-p8	F	53	toxic hepatitis	herbal medicine	0.71	≥60.00	156	128	142	99	3.9	14.0	138	0	0.06	0
Ajou-p9	M	42	toxic hepatitis	herbal medicine	0.75	≥60.00	213	35	80	NA	NA	NA	NA	0	3.074	0
Ajou-p10	M	41	toxic hepatitis	Medication	0.81	≥60.00	279	46	58	285	5.1	13.1	250	0	0.186	0
Ajou-p11	F	65	toxic hepatitis	Medication	0.6	≥60.00	155	17	10	8	6.9	13.2	100	0	0.272	0
Ajou-p12	M	62	toxic hepatitis	herbal medicine	0.84	≥60.00	146	39	70	134	4.6	15.0	109	0	0.824	0
Ajou-p13	F	50	toxic hepatitis	Medication	0.56	≥60.00	155	382	91	253	4.6	11.9	157	0	0.452	0
Ajou-p14	F	67	toxic hepatitis	herbal medicine	0.65	≥60.00	195	194	199	238	5.7	14.0	172	0	0.206	0
Ajou-p15	M	68	toxic hepatitis	herbal medicine	1.09	≥60.00	95	54	60	47	8.1	11.6	307	0	0.208	0
Ajou-p16	F	47	toxic hepatitis	herbal medicine	0.57	≥60.00	200	71	59	89	3.4	11.5	167	0	0.11	0
Ajou-p17	M	55	toxic hepatitis	herbal medicine	1.05	≥60.00	193	117	171	38	8.0	14.2	179	0	0.56	0
Ajou-p18	M	40	toxic hepatitis	herbal medicine	0.78	≥60.00	207	18	22	144	5.6	15.4	175	0	0.706	0
Ajou-p19	F	52	toxic hepatitis	Medication	0.53	≥60.00	234	20	26	44	NA	NA	NA	0	0.546	0
Ajou-p20	F	56	toxic hepatitis	Medication	0.48	≥60.00	227	58	126	103	5.4	13.3	317	0	0.27	0
Ajou-n1	M	50	Mached control		0.7	≥60.00	203	20	17	18	5.4	14.3	197	42.267	0.182	0
Ajou-n2	F	51	Mached control		0.6	≥60.00	207	14	18	23	4.8	12.4	311	22.916	0.038	0
Ajou-n3	M	52	Mached control		0.9	≥60.00	161	15	13	15	3.7	14.7	169	0	0.062	0.062
Ajou-n4	F	50	Mached control		0.6	≥60.00	198	19	17	12	3.3	13.8	239	33.578	0.068	0
Ajou-n5	M	46	Mached control		0.9	≥60.00	156	19	17	36	5.2	15.0	169	10.183	0.194	0
Ajou-n6	M	56	Mached control		0.93	≥60.00	148	19	13	35	5.7	14.8	178	14.489	0.696	0
Ajou-n7	F	56	Mached control		0.68	≥60.00	184	25	20	21	4.8	13.0	228	35.389	0.576	0
Ajou-n8	F	49	Mached control		0.76	≥60.00	188	21	9	12	4.0	12.2	275	0	0.676	0
Ajou-n9	F	51	Mached control		0.64	≥60.00	189	19	14	9	4.8	13.4	171	0	0.214	0
Ajou-n10	F	51	Mached control		0.65	≥60.00	185	31	17	14	3.7	11.7	195	0	0.092	0
Ajou-n11	F	50	Mached control		0.6	≥60.00	186	16	13	37	6.7	12.2	213	0	0.14	0
Ajou-n12	F	50	Mached control		0.62	≥60.00	189	18	16	15	5.7	14.1	292	0	0.022	0
Ajou-n13	M	49	Mached control		0.97	≥60.00	169	14	12	14	5.2	15.1	291	52.377	0.168	0
Ajou-n14	F	51	Mached control		0.78	≥60.00	203	17	12	15	5.4	12.6	229	109.741	0.446	0
Ajou-n15	F	50	Mached control		0.69	≥60.00	199	16	10	6	4.8	12.4	195	0	0.094	0
Ajou-n16	F	52	Mached control		0.67	≥60.00	204	19	18	14	8.0	14.4	255	0	0.692	0
Ajou-n17	F	49	Mached control		0.76	≥60.00	171	17	12	11	5.9	12.9	271	0	0.386	0
Ajou-n18	F	50	Mached control		0.71	≥60.00	187	22	25	24	4.0	12.5	218	0	0.166	0.227
Ajou-n19	F	50	Mached control		0.7	≥60.00	187	16	19	21	5.2	13.7	246	0	0.168	0
Ajou-n20	F	49	Mached control		0.6	≥60.00	204	19	12	13	4.7	13.2	233	0	0.226	0

Table S3. Chip assay for PCR primer.

Gene	NFκB binding site	Primer 5′ to 3′
**CYP2E1**	chr10:135332150-135332159	F; TAA AGC TCA GGT ACC ACG G
	GGCAATTTCC	R; TAC CTC CAT TAA CTA ACC TT
**IL6**	chr7:22766746-22766755	F; GCC CCA CCC TCA CCC TCC AA
	GGGATTTTCC	R; CCT CAG ACA TCT CCA GTC CT
**IL1α**	chr2:113532776-113532785	F; AAG TTT GGA TGG GCA ACT GA
	GTGAGTTTCC	R; GGA GAT GCC TGA GAT ACC CA
**IL17α**	chr6:52039129-52039138	F; AGT GCT TGG CTC AGC ATT TA
	TTGAATTTCC	R; AAA CTT GCC ACC TCA TGC TA
**CCL2**	chr17:32582162-32582171	F; GTG TCT CGT CCT GAC CCC CT
	GGAAATCCAC	R; AGG GAT CTT CCA TGA GTG A

Table S4. Real-time PCR primer.

Gene name	Forward Primer	Reverse Primer
**Human**		
IL6	AAT TCG GTA CAT CCT CGA CGG	GGT TGT TTT CTG CCA GTG CC
CCL2	AGC AGC AAG TGT CCC AAA GA	TTG GGT TTG CTT GTC CAG GT
SLIT2	GTC AAT GAC CTA CTG GCC TCA TTC	TGA TTG CTA CAC ACC ATT TCG TTT C
CYP2E1	GAG CAC CAT CAA TCT CTG GAC C	CAC GGT GAT ACC GTC CAT TGT G
ROBO1	GGC CCC ACT CCC CCT GTT CG	TCC TCT TCT GGC GCA TCC GTA TCC
ROBO2	GTT TGT GTT GCG AGG AAC TAT CT	GTT TTG TCG GAA GTC ATC TCG TA
ROBO3	CAG TGT CCG ATG GAA GAA GG	GTC CAT CTC CTG CAC ATT GG
ROBO4	GAC ACT TGG CGT TCC ACC TC	AGA GCA AGG AGC GAC GAC AG
IL1α	TGT ATG TGA CTG CCC AAG ATG AAG	AGA GGA GGT TGG TCT CAC TAC C
IL17α	CGG ACT GTG ATG GTC AAC CTG A	GCA CTT TGC CTC CCA GAT CAC A
CXCL10	GGT GAG AAG AGA TGT CTG AAT CC	GTC CAT CCT TGG AAG CAC TGC A
GAPDH	GTC TCC TCT GAC TTC AAC AGC G	ACC ACC CTG TTG CTG TAG CCA A
**Mouse**		
Ii6	TAC CAC TTC ACA AGT CGG AGG C	CTG CAA GTG CAT CAT CGT TGT TC
Ccl2	GCT ACA AGA GGA TCA CCA GCA G	GTC TGG ACC CAT TCC TTC TTG G
Slit2	ACC GTC TGA GAT GTA TCC CTC C	GCT GAC AAG TCA TTG AAG GCA CC
Cyp2e1	AGG CTG TCA AGG AGG TGC TAC T	AAA ACC TCC GCA CGT CCT TCC A
Robo1	GGC AGC CTG TTG GAT TAT CCT C	GTT GGC GTG AAG GTA AAG GAC G
Robo2	CCA CCA TCC AAA CCT CAG GTC A	TCT GCC AGC TAT TGC TCA CCG A
Robo3	ATA CGG CAG ACC AAG CTT CC	ACC AGC GTG TAT TGA CCT TCA
Robo4	GTC ATT GCC AGT AGT GCT GTC C	AAT GGC GTC CTC GCT GGT GTA T
Il1α	ACA TCT TTG ACG TTT CAG AGG TT	ACG AAG ACT ACA GTT CTG CCA TT
Il17α	CAG ACT ACC TCA ACC GTT CCA C	TCC AGC TTT CCC TCC GCA TTG A
Cxcl10	ATC ATC CCT GCG AGC CTA TCC T	GAC CTT TTT TGG CTA AAC GCT TTC
Gapdh	CAT CAC TGC CAC CCA GAA GAC TG	ATG CCA GTG AGC TTC CCG TTC AG

Table S5. The siRNA sequences used in this study.

Gene	Sequence (5′-3′)
**Human siSLIT2**	#1; GAUGCAGUGAACGGCUAUATT<br>UAUAGCCGUUCACUGCAUCTT
	#2; CCUCACCUUAAUUCUUAGUTT<br>ACUAAGAAUUAAGGUGAGGTT
**Human siROBO4**	#1; GCCUGUGAGUUAGGAAAUATT<br>UAUUUCCUAACUCACGGGCTT
	#2; GCCAAGACUACGAGUUCAATT<br>UUGAACUCGUAGUCUUGGCTT
**Human sip65**	#1; CCUCCUUUCAGGAGAUGAATT<br>UUCAUCUCCUGAAAGGAGGTT
**Mouse siRobo1**	#1; GGAUUAAUAUGUCUCUGUUTT<br>AACAGAGACAUAUUAAUCCTT
	#2; GACGAUAGAAUGGUACAAATT<br>UUUGUACCAUUCUAUCGUCTT
**Mouse siRobo2**	#1; CUCGGAUGGAGCCAUUUAUTT<br>AUAAAUGGCUCCAUCCCGAGTT
	#2; CGCGAUUCCAUAUCAAUAATT<br>UUAUUGAUAUGGAAUCGCGTT
**Mouse siRobo4**	#1; GGCCAAGACUAUGAAUUCATT<br>UGAAUUCAUAGUCUUGGCCTT
	#2; GCUGCUACUAGGCAUUACUTT<br>AGUAAUGCCUAGUAGCAGCTT
**Mouse siCyp2e1**	#1; GUCCGAGACAGGAUGAAUATT<br>UAUUCAUCCUGUCUCGGACTT
	#2; GCUCAUGAGUUUGUUCAAUTT<br>AUUGAACAAACUCAUGAGCTT

Table S6. Source data supporting all figures and supplementary figures in the manuscript (see accompanying Excel file).
